# A midbrain–cortical circuit mediated by a claustrum neuronal ensemble orchestrates drug-paired context memory processing

**DOI:** 10.1172/JCI196944

**Published:** 2026-01-15

**Authors:** Ziheng Zhao, Yuhong He, Yang Liu, Quying Feng, Hee Young Kim, Yu Fan, Xiaowei Guan

**Affiliations:** 1 Department of Human Anatomy and Histoembryology, Nanjing University of Chinese Medicine, Nanjing, China.; 2 Department of Physiology, Yonsei University College of Medicine, Seoul, Korea.

**Keywords:** Neuroscience, Public Health, Addiction, Behavior, Memory

## Abstract

Drug-associated environmental cues can trigger drug-seeking behavior and precipitate relapse. In this study, we determined that the claustrum (CL) connects the ventral tegmental area (VTA) with the medial prefrontal cortex (mPFC), forming the VTA–CL–mPFC circuit. Using a methamphetamine (METH) conditioned place preference (CPP) model in male mice, we found that manipulating the VTA–CL–mPFC circuit or CL neuronal ensemble receiving projections from VTA and projecting to mPFC (^VTA–^CL^–mPFC^) could disrupt the retrieval of METH-paired context memory, resulting in the blockage of the acquisition of METH CPP in male mice. During the process, dopamine release and dopamine 1-like receptor–mediated activation of CL neurons were required for the retrieval of METH-induced reward memory in male mice. These findings reveal a midbrain–cortical circuit orchestrated by CL neurons that plays an essential role in the retrieval of drug-paired environmental cue memory.

## Abstract 2

Zhao et al. identified a midbrain-to-cortical circuit orchestrated by claustrum, which plays an essential role in the retrieval process of drug-paired environmental cue memory.

## Introduction

Drug exposure produces strong reward-associated memories, which contribute to drug addiction or dependence (1, 2). Besides the direct reward properties of drugs, drug-paired environmental cues play a critical role in the process of reward memory (3, 4). In individuals with substance use disorder, robust memories that link the euphoric effects of drugs with contextual environment are stored in their brains, retrieval of which could trigger drug-craving behaviors and even relapse. In animal research, the animals exhibit an obstinate preference to drug-exposed environment contexts in a conditioned place preference (CPP) behavioral paradigm (5, 6). The ventral tegmental area (VTA) plays a pivotal role in reward processing and reinforcement learning of drug abuse, primarily through dopaminergic signaling (7, 8). The medial prefrontal cortex (mPFC) is a high-level executive region for decision-making, reward, and motivation and is regarded as a key brain region involved in the process of drug-associated reward memory. It has been widely believed that both the VTA–mPFC pathway and VTA–nucleus accumbens (NAc)–mPFC circuit are the key pathways for storing or retrieving drug reward memories (9–11). However, these pathways cannot fully explain how the external information of drug-exposed contextual environment is integrated with reward properties of drugs.

The claustrum (CL) is a thin sheet-like subcortical brain structure buried between insula and putamen, densely connecting the cortical regions with other subcortical regions, serving as a hub for multisensory integration (12, 13). The CL is predominantly composed of glutamatergic neurons (CL^Glu^) (14). Recent evidence, including ours, highlights that the CL^Glu^ neurons are involved in the drug-induced reward (15–17). The VTA provides dopaminergic input to the CL, and the CL sends outputs to the mPFC (18–20). The CL^Glu^ neurons abundantly express dopamine D1 receptor (D1R) and dopamine D2 receptor (D2R) (21–23). Furthermore, the CL is a key region for storing, integrating, and relaying multisensory information, such as visual and auditory information, exerting its role in attention, consciousness, reward, and emotion control (13, 24–26). As such, we speculate that there is a subgroup of CL^Glu^ neurons connecting VTA to mPFC, forming a VTA–CL–mPFC circuit, which facilitates the storage and retrieval of drug reward–associated contextual memories.

In this study, the methamphetamine-induced (METH-induced) CPP model was established to assess the roles of CL^Glu^ neurons, especially the intermediary neuronal ensemble connecting VTA with mPFC (^VTA–^CL^–mPFC^), in the retrieval of METH reward-related contextual environmental memories. Here, we focus on the tyrosine hydroxylase–positive (TH^+^) neurons in the VTA (VTA^TH^). In parallel, the DA signaling along the VTA–CL–mPFC circuit involved in the retrieval process of METH-induced reward memory was explored in male mice.

## Results

### The VTA^TH^–CL^Glu^–mPFC circuit and the ^VTA–^CL^–mPFC^ neuronal ensemble.

**Figure 1:**
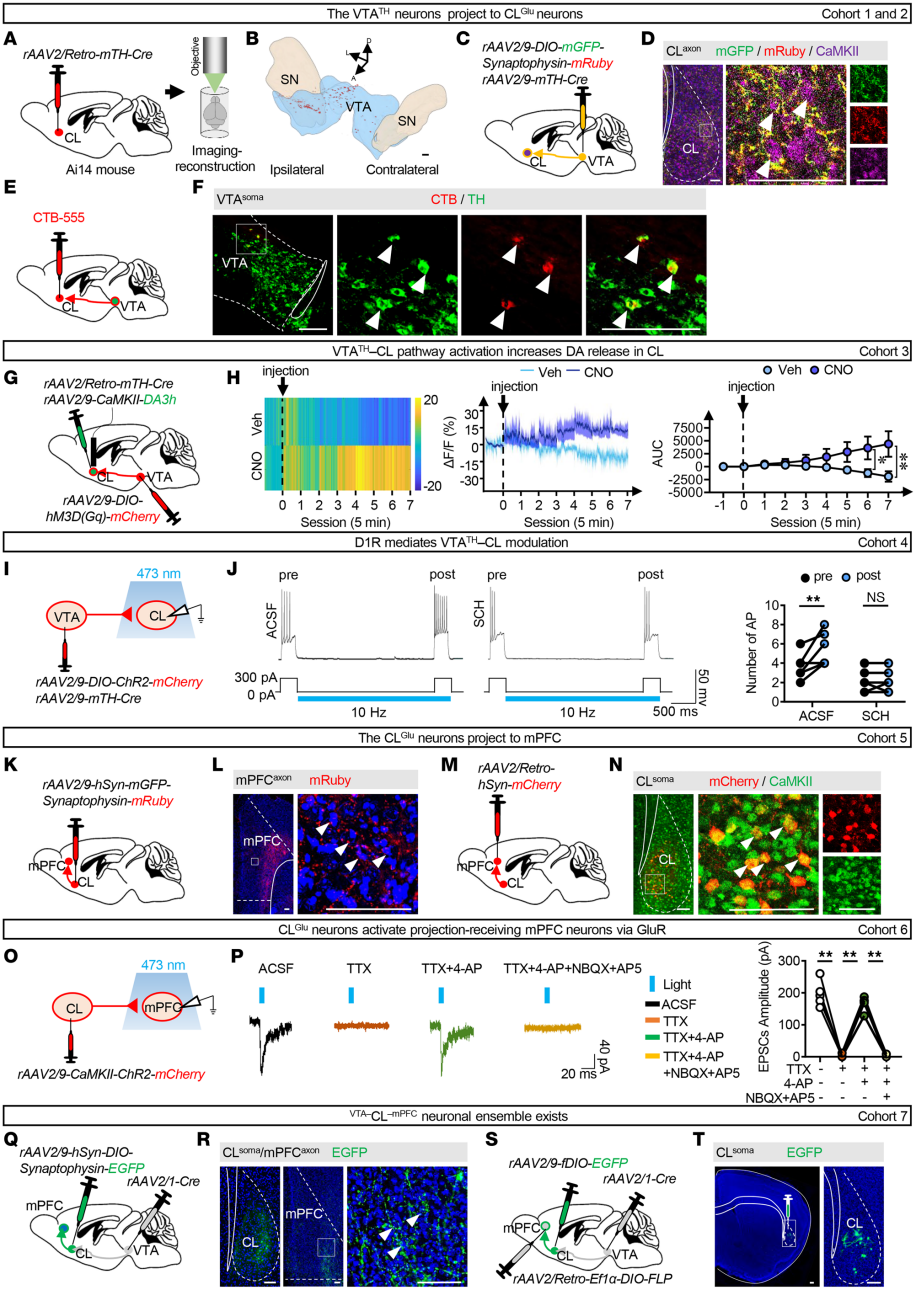
The VTA^TH^–CL^Glu^–mPFC circuit and the ^VTA–^CL^–mPFC^ neuronal ensemble. (**A**) Schematic of viral transfection. (**B**) Representative image of tdTomato^+^ neurons in VTA. (**C**) Schematic of viral transfection. (**D**) Representative images of mGFP^+^mRuby^+^ axon terminals around CL^Glu^ neurons. (**E**) Schematic of viral transfection. (**F**) Representative images of CTB-555^+^TH^+^ neurons in VTA. (**G**) Schematic of viral transfection. (**H**) Heatmap of DA3h fluorescence (left), quantification (middle), and AUC (right) of ΔF/F in CL. *n* = 4 mice/group. (**I**) Schematic of viral transfection. (**J**) Example traces and quantification of the number of APs. *n* = 6 cells from 6 mice/group. (**K**) Schematic of viral transfection. (**L**) Representative images of mRuby^+^ axon terminals in mPFC. (**M**) Schematic of viral transfection. (**N**) Representative images of CaMKII^+^mCherry^+^ neurons in CL. (**O**) Schematic of viral transfection. (**P**) Example traces and quantification of the amplitude of EPSCs. *n* = 5 cells from 6 mice/group. (**Q**) Schematic of viral transfection. (**R**) Representative images of EGFP^+^ neurons in CL and EGFP^+^ axon terminals in mPFC. (**S**) Schematic of viral transfection. (**T**) Representative images of EGFP^+^ neurons in CL. NS, *P* > 0.05, *P < 0.05, **P < 0.01. Two-way ANOVA with Šidák’s multiple-comparison test (**H** and **J**); 2-tailed paired *t* test (**P**). Scale bars: 100 μm (**B**, **D**, **F**, **L**, **N**, **R**, and **T**).

First, we performed retrograde labeling by injecting Retro-mTH-Cre virus into the CL in Ai14 mice to assess the projections from VTA to CL (Figure 1A). Whole-brain 3D imaging and reconstruction using fluorescent micro-optical sectioning tomography (fMOST) revealed that tdTomato^+^ somas were predominantly in the ipsilateral VTA, with a smaller population in the substantia nigra (SN) (Figure 1B and Supplemental Figure 1A; supplemental material available online with this article; https://doi.org/10.1172/JCI196944DS1). To elucidate the anatomical architecture of the VTA^TH^–CL pathway, we used anterograde tracing virus with mTH promoter–expressed Cre and Cre-dependent mGFP-Synaptophysin-mRuby, which were injected into VTA (Figure 1C and Supplemental Figure 1B). As shown in Figure 1D, the synaptophysin-labeled VTA^TH^ terminals were densely distributed around CaMKII^+^ CL^Glu^ neurons. Additionally, retrograde neuroanatomical tracer was injected into CL (Figure 1E and Supplemental Figure 1C), and cholera toxin subunit B–labeled (CTB-labeled) neurons were colabeled with TH in VTA (Figure 1F). These results confirmed the existence of the VTA^TH^–CL pathway.

Next, to investigate dopamine (DA) release from VTA to CL, the real-time DA3h signals within CL were measured by the DREADD (designer receptors exclusively activated by designer drugs) method in the VTA^TH^–CL pathway and fiber photometry recording above CL (Figure 1G and Supplemental Figure 1, D and E). The spontaneous action potentials (sAPs) increased following clozapine-*N*-oxide–mediated (CNO-mediated) activation of VTA^TH^ neurons (Supplemental Figure 1F). In parallel, the DA3h signals increased in CL from 30 to 35 minutes after CNO injection in freely moving mice compared with vehicle controls (Figure 1H). These findings indicate that the DA could be released into CL when CL-projecting VTA^TH^ neurons are activated.

Then, to determine whether CL-projecting VTA^TH^ terminals influence the activity of CL neurons, we utilized whole-cell patch clamp in CL slices to record the number of APs of CL neurons (Figure 1I). As shown in Figure 1J, the number of APs of CL neurons were increased when triggering the CL-projecting VTA^TH^ terminals by optogenetic activation, which was abolished by the following incubation of SCH-23390. As shown in Supplemental Figure 1, G and H, D1R was expressed both on the CL neurons and on the VTA^TH^ terminals within the CL. These results indicate that D1R is involved in the regulatory effects of VTA^TH^ terminals on the activity of CL neurons along the VTA^TH^–CL pathway.

Next, fMOST was performed by injecting an anterograde tracing virus into CL in WT mice to address the projections from CL to mPFC (Supplemental Figure 1I). The whole-brain reconstruction results revealed that CL sends dense projections to the ipsilateral mPFC (Supplemental Figure 1J). To further delineate the anatomical architecture of the CL–mPFC pathway, we injected a presynaptic localization virus into CL (Figure 1K and Supplemental Figure 1K). The mPFC exhibited a strong mRuby^+^ fluorescence signal, suggesting projections from CL to mPFC (Figure 1L). The anterograde monosynaptic tracking virus was injected into the CL to label mPFC neurons receiving projections from the CL (Supplemental Figure 1L). As shown in Supplemental Figure 1M, 87.5% of EGFP^+^ mPFC neurons were immunoreactive for CaMKII, indicating glutamatergic identity. Subsequently, as shown in Figure 1, M and N, and Supplemental Figure 1N, retrograde tracer virus was injected into mPFC, and immunoﬂuorescence analysis revealed that 91.8% of mCherry^+^ CL neurons were immunoreactive for CaMKII. These results support a glutamatergic projection from CL to mPFC, forming the CL^Glu^–mPFC pathway.

Then, to address the functional connectivity of the CL^Glu^–mPFC pathway, whole-cell patch clamp was conducted in acute mPFC slices, and light-evoked excitatory postsynaptic currents (EPSCs) were recorded by stimulating ChR2-expressing terminals with blue light (Figure 1O). EPSCs were suppressed by tetrodotoxin (TTX) and restored after mixing with 4-aminopyridine (4-AP), indicating that CL CaMKII-expressing neurons form monosynaptic connections with the downstream mPFC (Figure 1P). These EPSCs were abolished by coapplication of the specific glutamate receptor antagonist 2,3-dihydroxy-6-nitro-7-sulfamoyl-benzo(F)quinoxaline (NBQX) and d-2-amino-5-phosphonovaleric acid (AP5) (Figure 1P), confirming glutamatergic neurotransmission. These findings indicate that the CL^Glu^–mPFC pathway exhibits strong architectural and functional connections.

Finally, to verify the CL neuronal ensemble that connects VTA with mPFC (^VTA–^CL^–mPFC^ neurons), anterograde monosynaptic tracking virus was injected into VTA to label CL neurons receiving projections from VTA in WT mice (Figure 1Q). As shown in Figure 1R, EGFP-labeled somas were observed in CL, and EGFP^+^ axon terminals were observed in mPFC, indicating the existence of VTA–CL–mPFC projection. To further confirm this tripartite circuit, we employed an intersectional viral strategy by injecting the anterograde virus into VTA and the retrograde virus into mPFC, which successfully labeled a population of ^VTA–^CL^–mPFC^ neurons through the combination of fDIO, DIO-FLP, and Cre viral elements (Figure 1, S and T). These results demonstrate the existence of the ^VTA–^CL^–mPFC^ neuronal ensemble.

### The CL^Glu^ neurons are involved in the retrieval of METH-induced reward memory.

**Figure 2:**
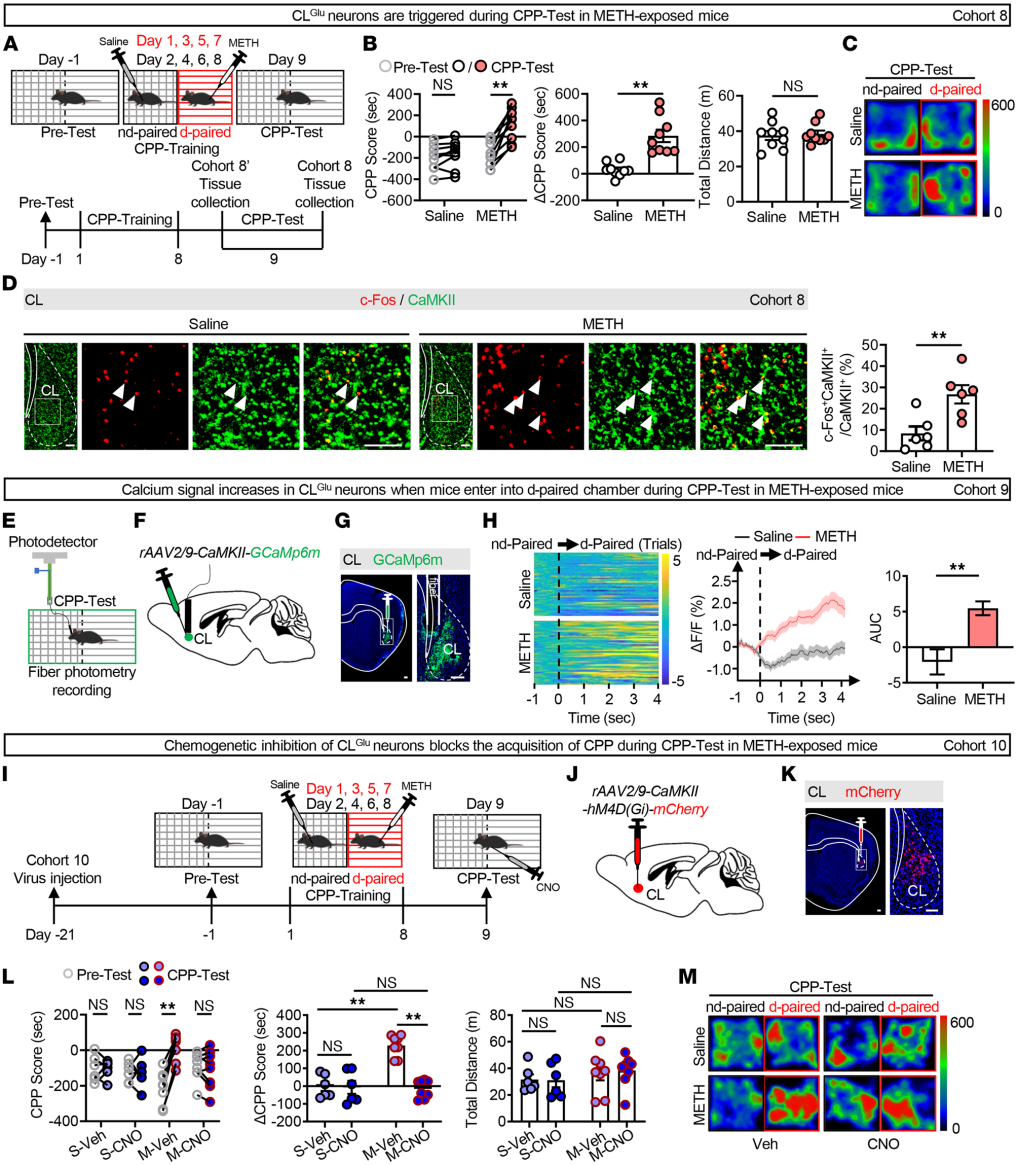
The CL^Glu^ neurons are involved in the retrieval of METH-induced reward memory. (**A**) Experimental design and timeline. (**B**) Analysis of CPP behavior of time spent in CPP apparatus. *n* = 9 mice/group. (**C**) Representative heatmap of time spent in CPP apparatus during CPP-Test. d-paired, drug-paired; nd-paired, non–drug-paired. (**D**) Immunofluorescence of c-Fos^+^CaMKII^+^ neurons in CL following METH CPP-Test. The percentage of c-Fos^+^CaMKII^+^ neurons in CL^Glu^. *n* = 6 mice/group. (**E**) Experimental design. (**F**) Schematic of viral transfection. (**G**) Representative images of virus injection and fiber implantation in CL. (**H**) Heatmap of GCaMp6m fluorescence (left), quantification (middle), and AUC (right) of ΔF/F in CL. *n* = 6 mice/group. (**I**) Experimental design and timeline. (**J**) Schematic of viral transfection. (**K**) Representative images of virus injection in CL. (**L**) Analysis of CPP behavior of time spent in CPP apparatus. S-Veh/S-CNO group, *n* = 6 mice/group; M-Veh/M-CNO group, *n* = 8 mice/group. (**M**) Representative heatmap of time spent in CPP apparatus during CPP-Test. NS, *P* > 0.05, **P < 0.01. Two-way ANOVA with Šidák’s multiple-comparisons test (**B** and **L**); 2-tailed unpaired *t* test (**B, D**, and **H**). Scale bars: 100 μm (**D**, **G**, and **K**).

To assess the role of CL in METH-associated reward behavior, a CPP procedure was conducted in male mice (Figure 2A). All male mice underwent CPP-Training (days 1–8). On day 9, 1 subset was euthanized for brain collection without the CPP-Test (forming saline′ and METH′ groups), while another subset underwent the CPP-Test (forming saline and METH groups) (Figure 2A). Male mice exposed to METH showed higher CPP-Test and ΔCPP scores compared with saline-exposed controls (Figure 2, B and C, and Supplemental Figure 2A), indicating successful acquisition of METH-induced CPP. There was no difference in total distance traveled between saline- and METH-exposed male mice, indicating similar locomotive ability between the 2 groups of mice (Figure 2, B and C).

Here, c-Fos, NeuN, CaMKII, and GAD67 were used as markers for activated neurons, total neurons, glutamatergic neurons, and GABAergic neurons, respectively. METH-exposed male mice increased the percentage of c-Fos^+^ neurons in the CL, specifically in those colabeled with CaMKII but not in those colabeled with GAD67, compared with controls (Figure 2D and Supplemental Figure 2, B and C). Moreover, the percentage of activated neurons or CaMKII^+^ neurons in CL was positively correlated with the ΔCPP score in METH-exposed male mice (Supplemental Figure 2, D and E), indicating an activation of CL^Glu^ neurons during the CPP-Test in METH-exposed male mice. In cohort 8′ mice, there was no obvious change in the neuronal activity of CL before the CPP-Test in METH-exposed male mice (Supplemental Figure 2F).

To further assess in vivo neuronal activity of CL^Glu^, the real-time calcium signals of CL^Glu^ neurons were recorded during the METH CPP-Test (Figure 2, E–G). The “zero” point was set when the bodies of the mice fully entered the METH-paired chamber from the non–drug-paired chamber in the CPP apparatus. Within the initial 4 seconds entering the METH-paired chamber from the non–drug-paired chamber, the calcium signals were notably elevated in METH-exposed male mice compared with those observed in saline-exposed male mice (Figure 2H). Conversely, within the initial 4 seconds after entering into the non–drug-paired chamber from the METH-paired chamber, the calcium signals were decreased in METH-exposed male mice compared with those observed in saline-exposed male mice (Supplemental Figure 2G). These results suggest that CL^Glu^ neurons exhibit an activated response during the retrieval, rather than the encoding, of METH-induced reward memory in male mice.

Next, to determine whether CL^Glu^ neuronal activity is required for the retrieval of METH CPP memory, we performed the hM4Di-DREADD method in CL of WT male mice (Figure 2, I and J). CNO was administered 30 minutes before the CPP-Test to suppress the activity of CL^Glu^ neurons, and the vehicle injection (vehicle-treated group) served as control. Post hoc observation of mCherry fluorescence confirmed the transgene expression in CL (Figure 2K). As shown in Supplemental Figure 2H, immunofluorescence analysis revealed high transfection efficiency across the 4 groups in CL, and CNO treatment reduced the activity of CL^Glu^ neurons in both saline- and METH-exposed male mice compared with the control vehicle treatment groups, validating the efficacy of chemogenetic inhibition. Additionally, whole-cell patch clamp in brain slices confirmed that bath application of CNO (10 μM) markedly suppressed the spiking activity of hM4Di-expressing CL^Glu^ neurons (Supplemental Figure 2I). Behaviorally, CNO-mediated chemogenetic inhibition of CL^Glu^ neurons attenuated METH CPP memory retrieval in METH-exposed male mice, as shown by lower CPP and ΔCPP scores. In contrast, CNO had no effect on saline-exposed male mice. ΔCPP scores were higher in vehicle-treated METH-exposed versus vehicle-treated saline-exposed male mice, indicating virus treatment did not alter METH CPP acquisition. Additionally, total distance traveled did not differ among the 4 male mice groups (Figure 2, L and M, and Supplemental Figure 2J). Taken together, these results indicate that CL^Glu^ neurons play a necessary role in the retrieval of METH-induced reward memory.

### The ^VTA–^CL^–mPFC^ neuronal ensemble mediates the retrieval of METH-induced reward memory.

**Figure 3:**
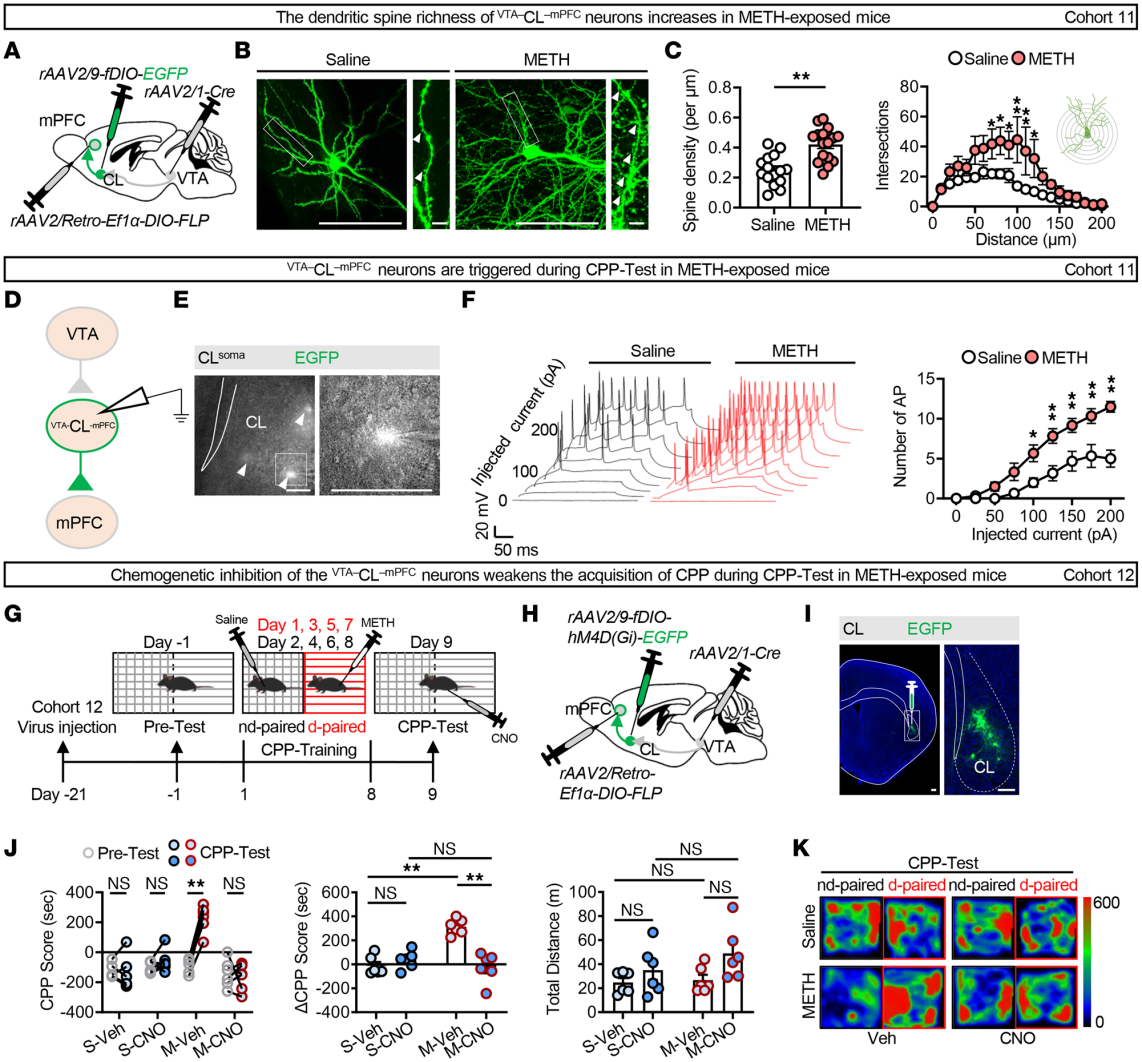
The ^VTA–^CL^–mPFC^ neuronal ensemble mediates the retrieval of METH-induced reward memory. (**A**) Schematic of intersectional viral transfection. (**B**) Representative images of dendritic spines in ^VTA–^CL^–mPFC^ neurons. (**C**) Sholl analysis of spine density (left panel). *n* = 15 spines from 8 saline-exposed mice, and *n* = 16 spines from 4 METH-exposed mice. The quantification of dendritic intersection (right panel). Saline group, *n* = 8 mice; METH group, *n* = 4 mice. (**D**) Schematic of patch-clamp viral transfection. (**E**) Representative images of virus injection and whole-cell patch clamp in CL. White arrowheads indicate ^VTA–^CL^–mPFC^ neurons. (**F**) The spike sample traces and the number of APs of ^VTA–^CL^–mPFC^ neurons under whole-cell patch clamp configuration. *n* = 6 cells from 3 mice/group. (**G**) Experimental design and timeline. (**H**) Schematic of viral transfection. (**I**) Representative images of virus injection in CL. (**J**) Analysis of CPP behavior of time spent in CPP apparatus. *n* = 6 mice/group. (**K**) Representative heatmap of time spent in CPP apparatus during CPP-Test. NS, *P* > 0.05, *P < 0.05, **P < 0.01. Two-way ANOVA with Šidák’s multiple-comparison test (**C**, **F**, and **J**); 2-tailed unpaired *t* test (**C**). Scale bars: 100 μm (**B**, **E**, and **I**), 10 μm (**B**, zoom).

To determine the role of the ^VTA–^CL^–mPFC^ neuronal ensemble in the retrieval of METH-induced reward memory, the same viral strategy as described for Figure 1S was used to label ^VTA–^CL^–mPFC^ neurons in male mice. As shown in Figure 3, A–C, METH-exposed male mice displayed greater spine density and complexity. As shown in Figure 3, D–F, the ^VTA–^CL^–mPFC^ neuronal ensemble exhibited an increase in the number of APs in METH-exposed male mice following the CPP-Test compared with saline controls. These results suggest that the ^VTA–^CL^–mPFC^ neurons are activated in METH-exposed male mice.

To examine the role of ^VTA–^CL^–mPFC^ neurons in the retrieval of METH CPP memory, we used a triple-virus chemogenetic strategy in male mice that selectively inhibited the EGFP^+^^VTA–^CL^–mPFC^ neurons by CNO (Figure 3, G–I). During the CPP-Test period, the ΔCPP score was much higher in vehicle-treated METH-exposed versus vehicle-treated saline-exposed male mice. However, CNO treatment reduced the preference for the METH-paired chamber in METH- but not saline-exposed male mice, as indicated by lower CPP and ΔCPP scores without affecting motor ability (Figure 3, J and K, Supplemental Figure 3A). These results demonstrate that the ^VTA–^CL^–mPFC^ ensemble is involved in the retrieval of METH-induced reward memory.

### The VTA^TH^–CL pathway contributes to the retrieval of METH-induced reward memory.

**Figure 4:**
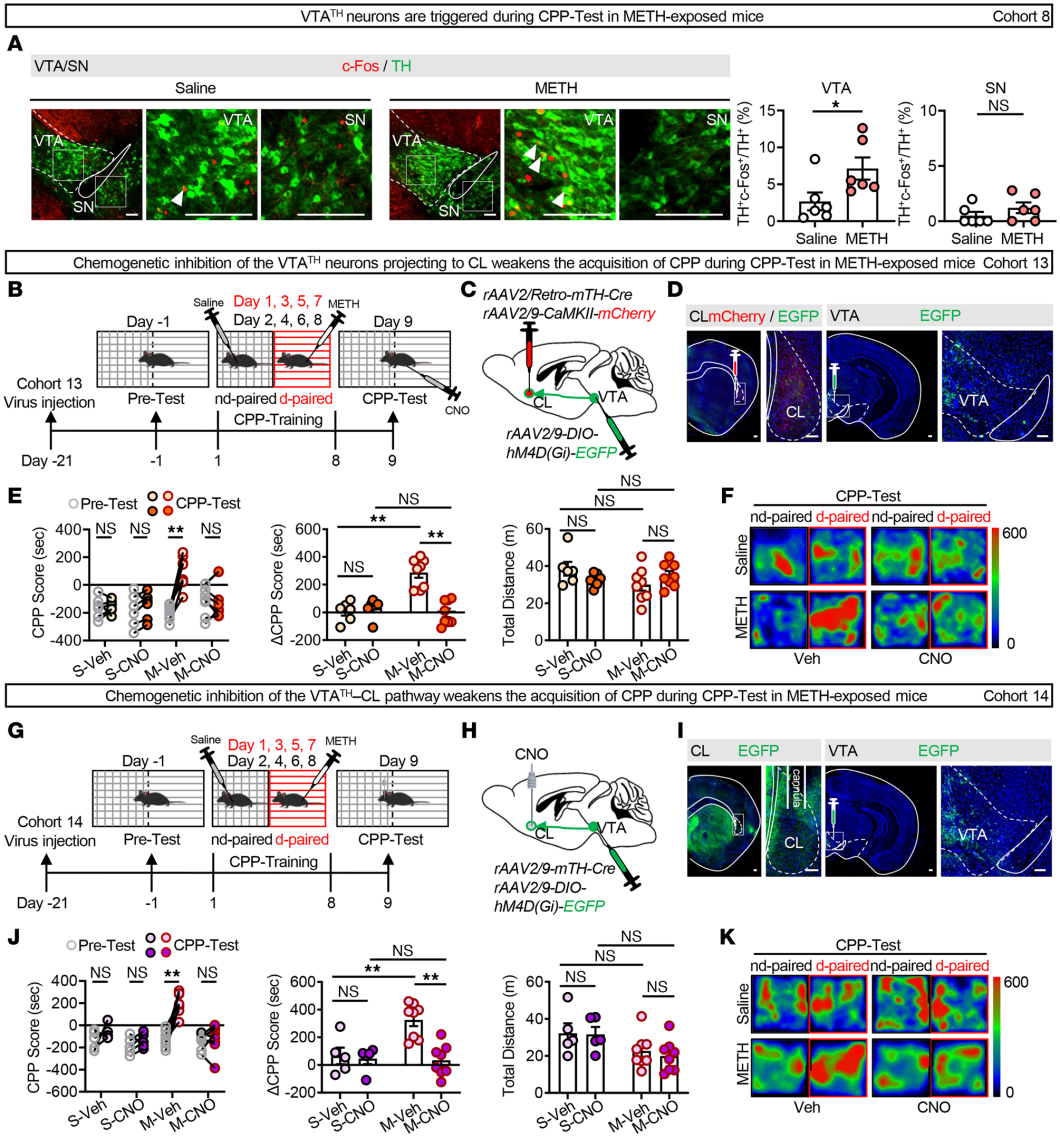
The VTA^TH^–CL pathway contributes to the retrieval of METH-induced reward memory. (**A**) Immunofluorescence for c-Fos^+^TH^+^ neurons in VTA and SN following the METH CPP-Test. The percentage of c-Fos^+^TH^+^ neurons in VTA^TH^ or SN^TH^. *n* = 6 mice/group. (**B**) Experimental design and timeline. (**C**) Schematic of viral transfection. (**D**) Representative images of virus injection in VTA or CL. (**E**) Analysis of CPP behavior of time spent in CPP apparatus. S-Veh/S-CNO group, *n* = 6 mice/group; M-Veh/M-CNO group, *n* = 8 mice/group. (**F**) Representative heatmap of time spent in CPP apparatus during CPP-Test. (**G**) Experimental design and timeline. (**H**) Schematic of viral transfection. (**I**) Representative images of cannula implantation in CL and virus injection in VTA. (**J**) Analysis of CPP behavior of time spent in CPP apparatus. S-Veh/S-CNO group, *n* = 5 mice/group; M-Veh/M-CNO group, *n* = 8 mice/group. (**K**) Representative heatmap of time spent in CPP apparatus during CPP-Test. NS, *P* > 0.05, *P < 0.05, **P < 0.01. Two-way ANOVA with Šidák’s multiple-comparison test (**E** and **J**); 2-tailed unpaired *t* test (**A**). Scale bars: 100 μm (**A**, **D**, and **I**).

Neuronal activation profiles in TH^+^ neurons of VTA and SN were performed. As shown in Figure 4A, the percentage of c-Fos^+^ neurons in TH^+^ neurons was increased in the VTA but not in the SN of METH-exposed male mice compared with the saline-exposed male mice, indicating that the VTA^TH^ neurons were activated during the METH CPP-Test.

To address the role of VTA^TH^ neurons projecting to CL in the retrieval of METH-induced reward memory, CL^Glu^ neurons and CL-projecting VTA^TH^ neurons were labeled in WT male mice (Figure 4, B–D). Supplemental Figure 4, A and B, presents the viral transfection rate in TH^+^ neurons and the viral functional efficiency. As shown in Figure 4, E and F, and Supplemental Figure 4C, CNO treatment suppressed the preference for METH-paired chamber in METH-exposed male mice, as indicated by lower CPP and ΔCPP scores, but did not affect these scores in saline-exposed male mice. There was no difference in the total distance traveled among the 4 groups of male mice.

Next, we validated the role of the VTA^TH^–CL pathway in the retrieval of METH-induced reward memory (Figure 4, G–I). As shown in Supplemental Figure 4D, local infusion of CNO treatment in CL efficiently reduced the percentage of c-Fos^+^ neurons in CL^Glu^ neurons in METH-exposed male mice. As shown in Figure 4, J and K, and Supplemental Figure 4E, suppressing the VTA^TH^ terminals in CL inhibited the acquisition of METH CPP, as indicated by lower CPP and ΔCPP scores in METH- but not saline-exposed male mice during the CPP-Test, while leaving their motor capabilities intact. These results suggest that the VTA^TH^–CL pathway contributes to the retrieval of METH-induced reward memory.

### The CL^Glu^–mPFC pathway contributes to the retrieval of METH-induced reward memory.

**Figure 5:**
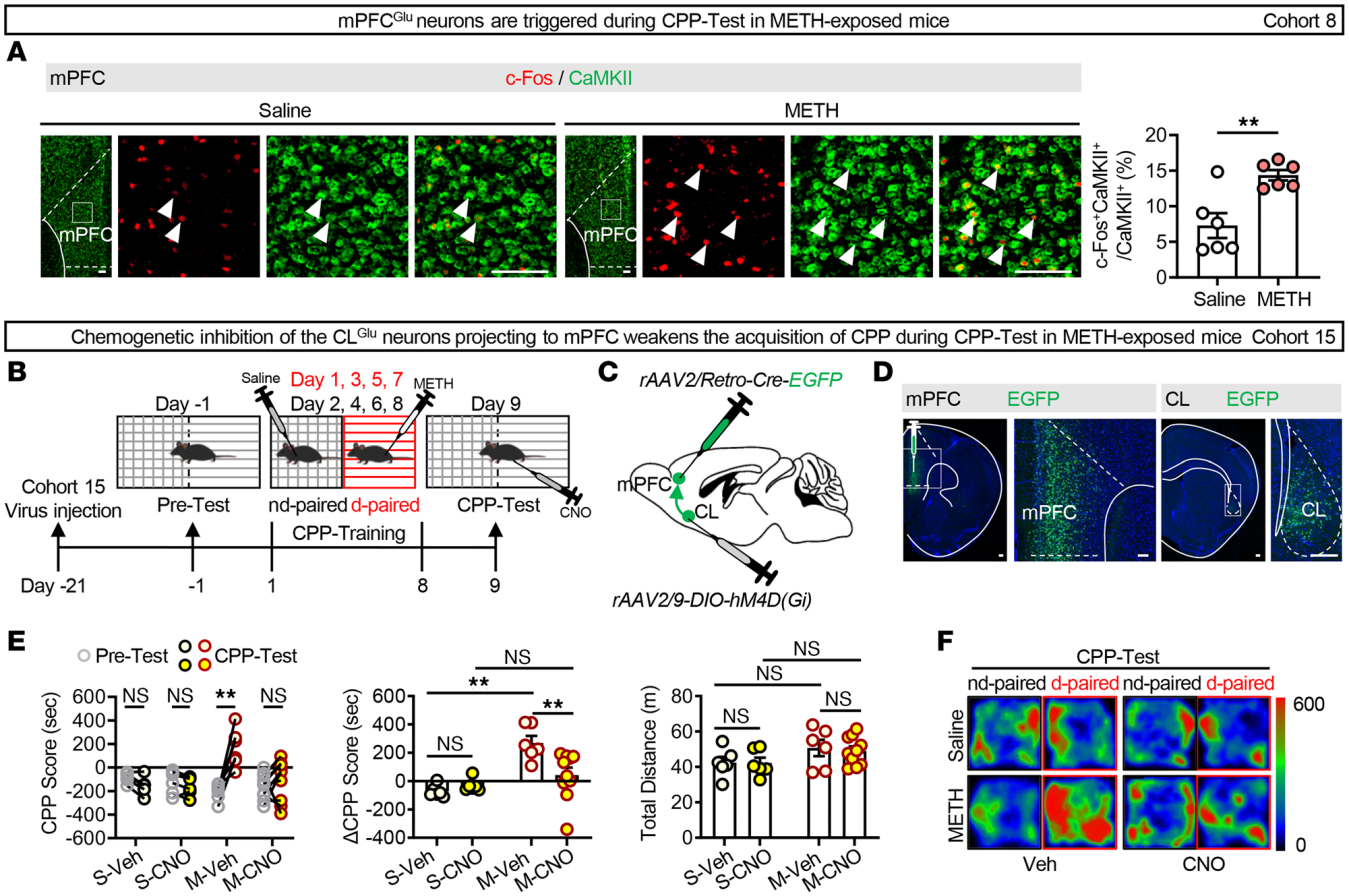
The CL^Glu^–mPFC pathway contributes to the retrieval of METH-induced reward memory. (**A**) Immunofluorescence of c-Fos^+^CaMKII^+^ neurons in mPFC following the METH CPP-Test. The percentage of c-Fos^+^CaMKII^+^ neurons in mPFC^Glu^. *n* = 6 mice/group. (**B**) Experimental design and timeline. (**C**) Schematic of viral transfection. (**D**) Representative images of virus injection in mPFC and retrograde cell body in CL. (**E**) Analysis of CPP behavior of time spent in CPP apparatus. S-Veh/S-CNO/M-Veh group, *n* = 6 mice/group; M-CNO group, *n* = 10 mice/group. (**F**) Representative heatmap of time spent in CPP apparatus during CPP-Test. NS, *P* > 0.05, **P < 0.01. Two-way ANOVA with Šidák’s multiple-comparison test (**E**); 2-tailed unpaired *t* test (**A**). Scale bars: 100 μm (**A** and **D**).

As shown in Figure 5A, immunoﬂuorescence analysis showed that METH-exposed male mice exhibited stable elevation of c-Fos expression in mPFC^Glu^ neurons during the CPP-Test. mPFC neurons projecting to CL were labeled to assess their role in the retrieval of METH-induced reward memory (Figure 5, B–D). The immunofluorescence analysis confirmed comparable viral transfection specificity across all groups, and CNO treatment markedly reduced the activity of coexpression of EGFP in CL^Glu^ neurons (Supplemental Figure 5A). As shown in Figure 5, E and F, and Supplemental Figure 5B, CNO treatment during the CPP-Test period weakened the acquisition of METH CPP in METH-exposed male mice, as evidenced by the decreased CPP and ΔCPP scores, suggesting that the CL^Glu^–mPFC pathway contributes to the retrieval of METH-induced reward memory.

### DA signaling along the VTA^TH^–CL pathway is crucial for the retrieval of METH-induced reward memory.

**Figure 6:**
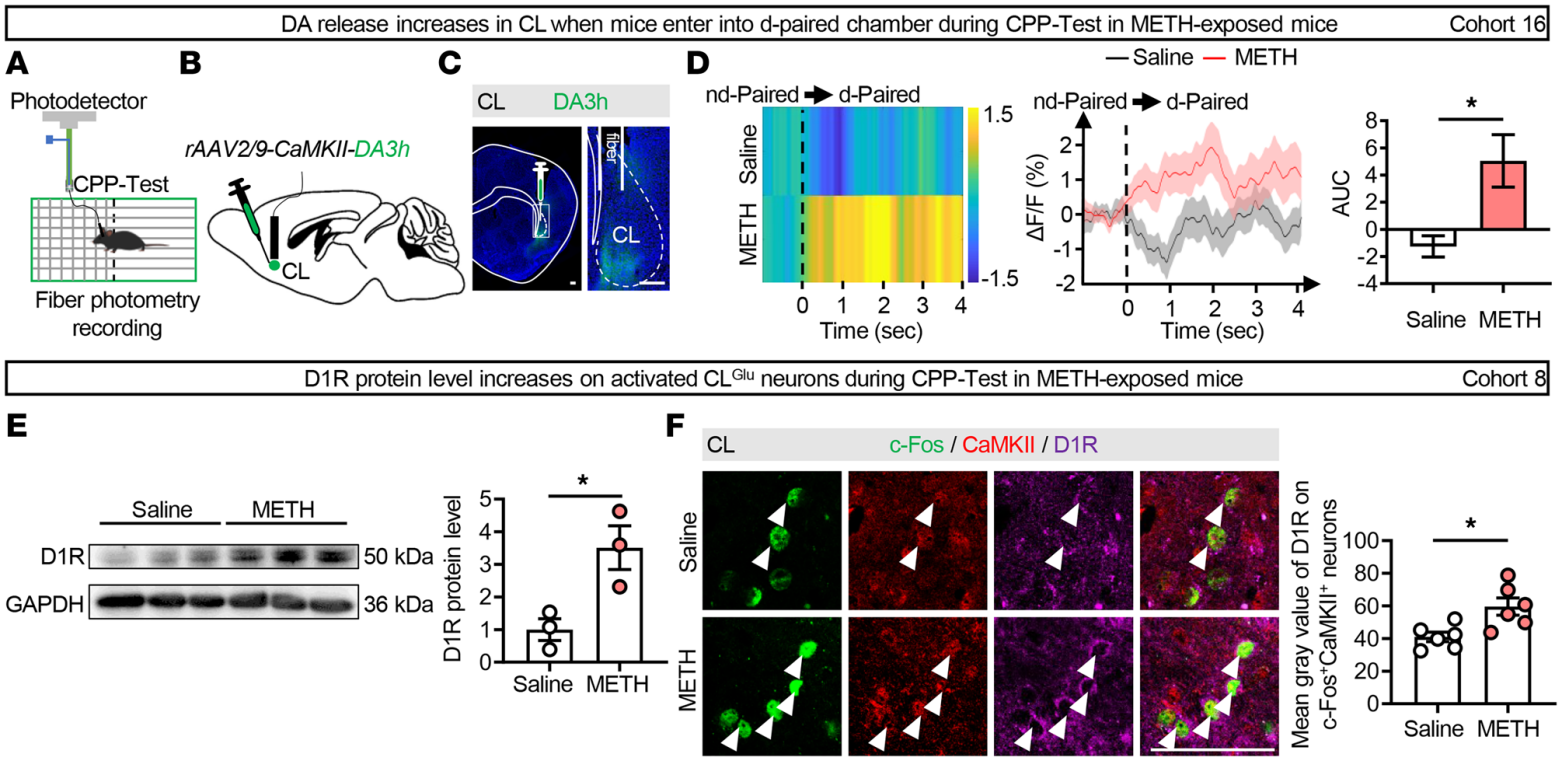
The DA release and D1R levels on CL^Glu^ neurons increase during the retrieval of METH-induced reward memory. (**A**) Experimental design. (**B**) Schematic of viral transfection. (**C**) Representative images of virus injection and fiber implant in CL. (**D**) Heatmap of DA3h fluorescence (left), quantification (middle), and AUC (right) of ΔF/F in CL. *n* = 5 mice/group. (**E**) The protein levels of D1R in CL. The protein levels of D1R following the METH CPP-Test. *n* = 3 mice/group. (**F**) Immunofluorescence of c-Fos^+^CaMKII^+^D1R^+^ neurons in CL. Mean gray value of D1R^CaMKII^ following METH CPP-Test. *n* = 6 mice/group. *P < 0.05. Two-tailed unpaired *t* test (**D**–**F**). Scale bars: 100 μm (**C** and **F**).

To measure the real-time DA release in CL during the METH CPP-Test in male mice, DA3h virus was injected into CL for recording DA release by fiber photometry (Figure 6, A–C). As shown in Figure 6D, the DA3h signals in CL were elevated when METH-exposed male mice fully entered into the METH-paired chamber during the CPP-Test. The protein levels of D1R and D2R were then measured in CL. As shown in Figure 6E and Supplemental Figure 6A, the protein levels of D1R but not D2R were higher in the CL of METH-exposed male mice compared with that of control male mice. The mean gray value of D1R expression on CL c-Fos and CaMKII double-positive neurons was higher in METH-exposed male mice than in control male mice (Figure 6F), suggesting increased D1R levels on activated CL^Glu^ neurons during the METH CPP-Test. These results indicate that both DA release and D1R levels are elevated in CL during the retrieval of METH-induced reward memory.

**Figure 7:**
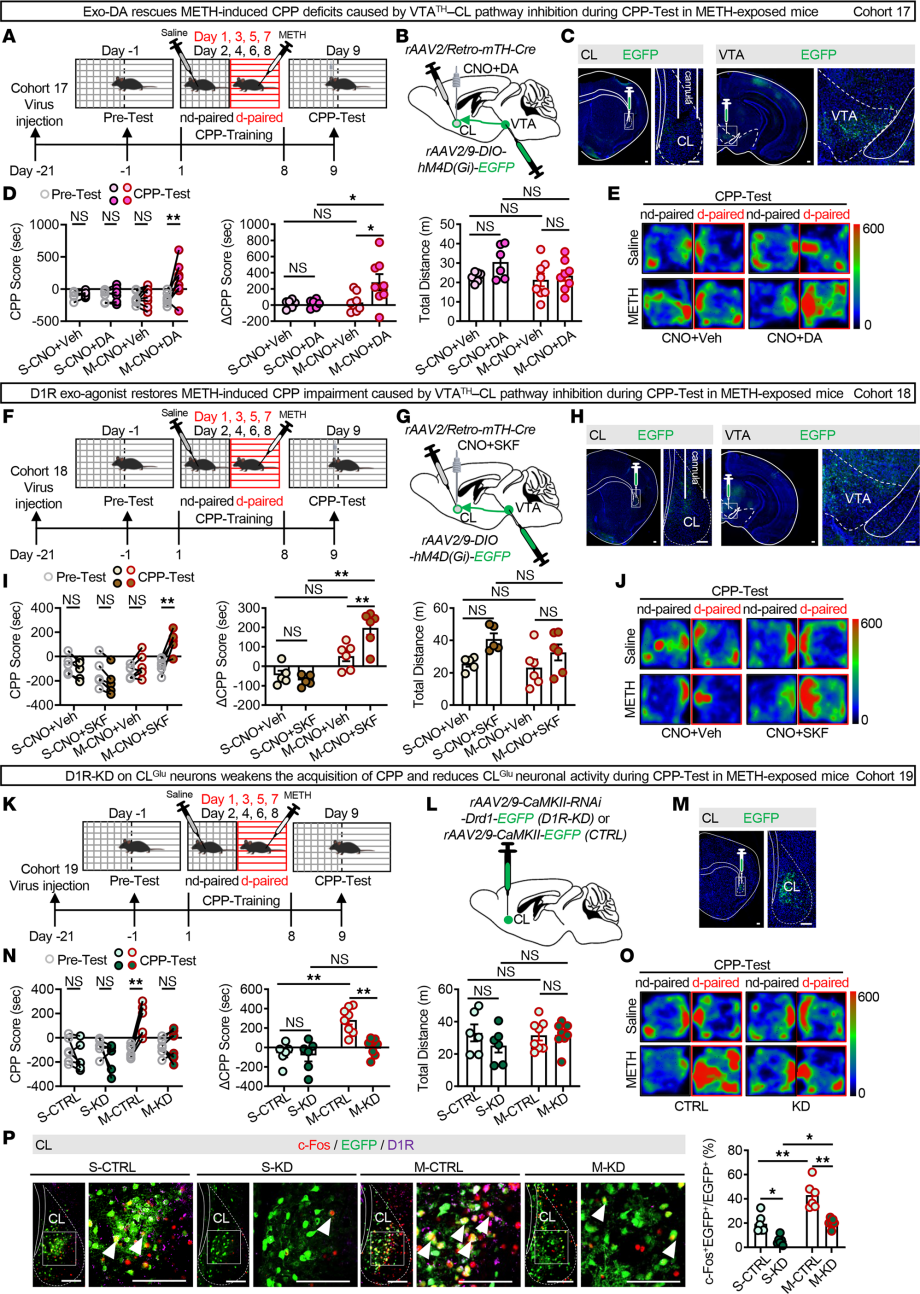
DA signaling along the VTA^TH^–CL pathway is crucial for the retrieval of METH-induced reward memory. (**A**) Experimental design and timeline. (**B**) Schematic of viral transfection. (**C**) Representative images of virus injection and cannula implantation in VTA or CL. (**D**) Analysis of CPP behavior of time spent in CPP apparatus. S-CNO+Veh/S-CNO+DA group, *n* = 6 mice/group; M-CNO+Veh/M-CNO+DA group, *n* = 8 mice/group. (**E**) Representative heatmap of time spent in CPP apparatus during CPP-Test. (**F**) Experimental design and timeline. (**G**) Schematic of viral transfection. (**H**) Representative images of virus injection and cannula implantation in VTA or CL. (**I**) Analysis of CPP behavior of time spent in CPP apparatus. S-CNO+Veh/S-CNO+SKF group, *n* = 5 mice/group; M-CNO+Veh/M-CNO+SKF group, *n* = 6 mice/group. (**J**) Representative heatmap of time spent in CPP apparatus during CPP-Test. (**K**) Experimental design and timeline. (**L**) Schematic of viral transfection. (**M**) Representative images of virus injection in CL. (**N**) Analysis of CPP behavior and heatmap of time spent in CPP apparatus. S-CTRL/S-KD group, *n* = 6 mice/group; M-CTRL/M-KD group, *n* = 8 mice/group. (**O**) Representative heatmap of time spent in CPP apparatus during CPP-Test. (**P**) Immunofluorescence of c-Fos^+^D1R^+^EGFP^+^ neurons in CL following CPP-Test. The percentage of c-Fos^+^EGFP^+^ neurons in EGFP^+^ neurons of CL. *n* = 6 mice/group. NS, *P* > 0.05, *P < 0.05, **P < 0.01. Two-way ANOVA with Šidák’s multiple-comparison test (**D**, **I**, **N**, and **P**). Scale bars: 100 μm (**C**, **H**, **M**, and **P**).

Next, we assessed the role of DA and D1R in the VTA^TH^–CL pathway in the retrieval of METH-induced reward memory. To assess whether local application of DA in the CL could rescue memory retrieval when the VTA^TH^–CL pathway was inhibited, DA and CNO were coinfused into the CL 5 minutes before the CPP-Test (Figure 7, A–C). As shown in Supplemental Figure 7A, under CNO-induced inhibition of VTA^TH^–CL projections, infusion of DA increased c-Fos expression on CL^Glu^ neurons in both saline- and METH-exposed male mice relative to vehicle controls. As shown in Figure 7, D and E, and Supplemental Figure 7B, the coinfusion of DA and CNO into CL enhanced the preference for the METH-paired chamber in METH-exposed male mice, as shown by increased CPP and ΔCPP scores during the CPP-Test period. To assess whether the activation of local D1R in CL could rescue memory retrieval when the VTA^TH^–CL pathway was inhibited, selective SKF-38393 (SKF) and CNO were coinfused into the CL 5 minutes before the CPP-Test (Figure 7, F–H). As shown in Supplemental Figure 7C, under CNO-induced inhibition of VTA^TH^–CL projections, infusion of SKF increased c-Fos expression on CL^Glu^ neurons in both saline- and METH-exposed male mice relative to vehicle controls. As shown in Figure 7, I and J, and Supplemental Figure 7D, the coinfusion of SKF and CNO into CL enhanced the preference for the METH-paired chamber in METH-exposed male mice, as indicated by increased CPP and ΔCPP scores during the CPP-Test period. These results indicate that DA signaling along the VTA^TH^–CL pathway is involved in the retrieval of METH-induced reward memory.

Finally, to explore the role of D1R levels on CL^Glu^ neurons in the retrieval of METH-induced reward memory, we knocked down the D1R on CL^Glu^ neurons in WT male mice by infusing the D1R-KD virus into CL (Figure 7, K–M). As shown in Supplemental Figure 8A, D1R-KD virus reduced D1R levels in CL compared with control virus, indicating effectivity of the virus. As shown in Supplemental Figure 8B, there was similar transfection efficiency among the 4 groups of male mice. During the CPP-Test period, D1R-KD virus treatment reduced CPP and ΔCPP scores in METH-exposed male mice but did not affect these scores in saline-exposed male mice (Figure 7, N and O, and Supplemental Figure 8C). As shown in Figure 7P, compared with corresponding control virus treatment male mice, the percentage of c-Fos^+^ neurons on EGFP-transfected neurons was much lower in both D1R-KD virus treatment saline- and METH-exposed male mice. These results indicate that the increased D1R expression on CL^Glu^ neurons contributes to their activation of CL^Glu^ neurons in METH-exposed mice.

## Discussion

Drug-related memories, which could be triggered by reexposure to drug-associated cues, contribute to drug-seeking behaviors (27, 28). In this study, we utilized the CPP paradigm to explore the retrieval of METH reward–associated context memory. Medina et al. posited that CL neuronal activity contributes to the acquisition, consolidation, and reconsolidation of memories in mice (29). Our METH CPP findings demonstrate that the CL is a key hub to relay reward information from the VTA to mPFC, which is orchestrated by the ^VTA–^CL^–mPFC^ neuronal ensemble. This ensemble plays a crucial role in the retrieval of the METH-induced reward memory along the VTA–CL–mPFC circuit. Along the circuit, DA signaling contributes to the activation of CL neurons, which is essential for the retrieval of METH-induced reward memory.

The CL is densely interconnected with widespread cortical areas, including prefrontal, somatosensory, visual, and motor cortices, and receives neuromodulatory inputs from subcortical areas, such as midbrain and thalamus (12, 13). Terem et al. reported that suppressing CL neurons reduces cocaine-driven behavior (30). Our previous study showed that the CL contributes to the cocaine susceptibility in cocaine-exposed mice in a region-specific manner (15). As such, we hypothesized that a neuronal ensemble of CL is involved in the drug-associated reward memory. Consistent with these reports, we found that CL serves as a hub connecting VTA^TH^ neurons to the mPFC, forming the VTA–CL–mPFC circuit, and that a CL neuronal ensemble connects VTA^TH^ neurons to the mPFC (^VTA–^CL^–mPFC^ neurons). In addition, we found that either the CL^Glu^ neurons or the ^VTA–^CL^–mPFC^ neuronal ensemble was activated in METH-exposed mice during the CPP-Test. Suppression of either CL^Glu^ neurons or the ^VTA–^CL^–mPFC^ neuronal ensemble impaired the retrieval of METH-induced reward memory in mice. These findings demonstrate that the ^VTA–^CL^–mPFC^ neuron ensemble plays an important role in drug-associated reward memory.

What role does the VTA–CL–mPFC circuit play in the retrieval of METH-induced reward memory? We found that disrupting either the VTA^TH^–CL or CL^Glu^–mPFC pathway impaired the METH-induced memory retrieval. The METH contextual conditioning process is mediated by distinct neural pathways with specialized functions. The well-established VTA–NAc–mPFC circuit, in which NAc serves as a critical relay node, is predominantly engaged by the direct pharmacological reinforcing effects of the drug itself (31, 32). By contrast, the CL operates as a higher-order sensory integration hub, with widespread connections across cortical and subcortical structures. This unique anatomical architecture endows the CL with a greater sensitivity for processing complex contextual environmental cues, such as those encountered in cocaine-paired contexts (29, 33, 34). We speculated that the VTA–NAc–mPFC circuit governs the primary rewarding properties of METH, whereas the VTA–CL–mPFC circuit may contribute to the establishment of associative memory linking drug reward effects with contextual cues. The CPP is a widely used behavioral paradigm to assess reward-related memories that contains training and test phases (35, 36). During the training period of METH CPP, animals establish an associative memory linking the rewarding effects of METH with the surrounding environment (METH-paired chamber). During the METH CPP test period, upon reexposure to the METH-paired context that recalls the METH-related reward memories, animals acquired a preference for the previously drug-associated environment despite the absence of the drug. DA-releasing neurons of the VTA have central roles in reward-related behaviors (37). Here, we found that VTA^TH^ neurons were activated during the METH CPP-Test. Suppression of the CL-projecting VTA^TH^ terminals attenuated METH-induced reward memory retrieval, suggesting that environmental cues can drive METH-associated memories, at least partially through recruiting the VTA^TH^–CL pathway. The CL projects to frontal cortical regions that are critically involved in encoding drug-related reward (17, 30). Emerging evidence implies that CL projections to the frontal cortices are involved in the cognitive tasks of drug-associated memories (30, 38). Another study reported that inhibition of claustral neurons projecting to the mPFC attenuated impulsive behaviors induced by METH exposure (39). Similar with these reports, we found that inhibition of mPFC-projecting CL^Glu^ neurons impairs the retrieval of METH-induced reward memory. Previous studies have shown that activation of CL neurons predominantly suppress mPFC neuronal activity through inducing strong and widespread feedforward inhibition (FFI) (18, 40). However, we found that during the retrieval of METH-induced reward memory, when the CL neurons were activated, the mPFC neurons were triggered parallely instead of being inactivated. We speculate that METH exposure may weaken the FFI exerted by CL on mPFC neurons, which might contribute to the observed activation of mPFC neurons.

As mentioned above, the VTA^TH^–CL pathway contributes to the retrieval of METH-induced reward memory. We next determined whether DA signaling along the pathway plays a role in this process. DA is well known to orchestrate reward learning through dynamic and temporally patterned release (41). We found that both DA release and D1R levels were increased in the CL of METH-exposed male mice. Either inhibition or knockdown of D1R impaired the METH-induced reward memory retrieval. Notably, D1R levels, rather than those of D2R, in the CL were increased during the METH CPP-Test. D1R activation in the mPFC has been shown to facilitate METH-induced hyperactivity (42), and intraperitoneal injection of a D1R inhibitor but not a D2R inhibitor before METH self-administration training could reduce drug-seeking behavior (43). Terem et al. demonstrated that D1R^+^ CL neurons are essential for acquiring cocaine-preferred behaviors, and stimulation of these neurons reinstates cocaine-seeking behavior in a context-dependent manner (30). In line with this, our previous study revealed that cocaine exposure activated CL^Glu^ and increased the expression of D1R, and the number of APs of CL neurons was reduced by the D1R antagonist SCH-23390 in mice (44). In this study, either exogenous DA administration or D1R activation in the CL reversed the impairment of METH-induced reward memory retrieval by suppressing the VTA^TH^–CL pathway. These results demonstrate that DA signaling along the VTA–CL pathway is involved in the retrieval of METH-associated memories.

There are several limitations to this study. First, the current findings did clarify whether the regulatory effects of DA release by VTA^TH^ on D1R and neuronal excitability of CL neurons are mediated through monosynaptic transmission or through accumulated volume transmission. Second, we found that D1R is expressed both on VTA^TH^ terminals (presynaptic D1R) and on CL neurons (postsynaptic D1R) along the VTA–CL pathway. Our current findings did not clarify whether the regulatory effects of a D1R antagonist infused into the CL on neuronal excitability of CL neurons are mediated through presynaptic or postsynaptic D1R. Third, a subset of VTA dopaminergic neurons is known to corelease glutamate with DA (45, 46). However, in this study, we did not explore the phenotype and role of coreleased glutamate along the VTA^TH^–CL pathway in the regulation of CL activity under either physiological or METH-induced pathological conditions.

## Methods

### Sex as a biological variable.

Only male mice were used in this study. Numerous studies have demonstrated that there are sex differences in the response to psychostimulants (47–49). We propose that these differences are partially due to the effects of the estrous cycle and hormonal fluctuations on neuronal excitability and behavioral outcomes. As such, to exclude the potential sex-specific effects of the VTA–CL–mPFC circuit on METH-induced reward memory, we only used male mice. Our previous study showed that METH changed the activity pattern of the brain in a sex-dependent manner (50). Therefore, we speculate that the current findings in male mice are not directly generalizable to female mice.

### Animals.

Male C57BL/6 WT mice (Changzhou Cavens Experimental Animal Co.) and Ai14-Cre mice (National Institute of Biological Sciences, Beijing, China), approximately 20–25 g and 8–10 weeks of age, were used in this study. All animals were housed at constant humidity (50% ± 10%) and temperature (24°C ± 2°C) with a 12-hour light/dark cycle (lights on at 8 am) and allowed free access to food and water in their home cages.

### Stereotaxic surgery.

Male mice were anesthetized with 2% isoflurane and placed on a stereotaxic apparatus (RWD). The eyes were covered with eye ointment (Cisen) to prevent drying. Following hair shaving and disinfection of the incision site using iodine and 70% medical alcohol, the scalp was incised to expose the skull. A 30% hydrogen peroxide solution was applied to the skull to dissolve connective tissue, which was then gently removed from the skull surface with cotton swabs. Small craniotomy holes (~1 mm diameter) were drilled with the aid of a microscope (RWD) for virus injection, or optic fiber or cannula embedding. A volume of 50, 100, or 200 nL virus was injected in CL, VTA, or mPFC per side, respectively. The injections were given over 5 minutes at a rate of 10–40 nL/min by an infusion pump (RWD) and left in place for 5 minutes. The stereotaxic coordinates utilized were as follows: CL (AP, +0.98 mm; ML, ±2.85 mm; DV, −3.85 mm), VTA (AP, −3.16 mm; ML, ±0.5 mm; DV, −4.1 mm), and mPFC (AP, +1.94 mm; ML, ±0.4 mm; DV, −2.45 mm). The mice with inaccurate location of viral injection, fiber optic implantation, or cannula implantation were excluded from the subsequent experiments in this study. Unless otherwise specified, all surgical procedures were performed bilaterally.

### Neuron label and virus trace.

All viruses were from BrainVTA, BrainCase, and GeneChem. Male mice were fixed in a stereotactic frame (RWD) under 2% isoflurane anesthesia, with the body temperature maintained at 37°C using a heating pad.

We examined the projection of the VTA^TH^–CL pathway of WT mice by unilaterally injecting the retrograde neuroanatomical tracer Cholera Toxin Subunit B 555 (CTB-555, CTB-02, 1 μg/μL, BrainVTA) into CL (cohort 2 mice). For anterograde tracing, we examined the projection pattern and synaptic targets of VTA^TH^ neurons of WT mice (cohort 2 mice) by unilaterally injecting a synaptophysin fused with red fluorescent protein (mRuby) rAAV2/9-hSyn-DIO-mGFP-T2A-Synaptophysin-mRuby-WPRE-hGH pA (PT-1244, 5.65 × 10^12^ vg/mL, BrainVTA) in a Cre-dependent manner mixed with mTH-Cre virus rAAV2/9-mTH-NLS-CRE-WPRE-SV40 pA (PT-0781, 5 × 10^12^ vg/mL, BrainVTA). This approach restricted the mRuby expression to the presynaptic terminals, allowing us to discriminate between putative synaptic targets and the axon of passage. Moreover, it showed the axons of VTA^TH^ neurons projecting to CL, and the terminals were observed with mRuby signal in CL.

We also examined the projection of the CL^Glu^–mPFC pathway of WT mice by unilaterally injecting the anterograde tracing virus rAAV2/9-hSyn-mGFP-T2A-Synaptophysin-mRuby-WPRE-hGH pA (PT-1243, 5.05 × 10^12^ vg/mL, BrainVTA) into CL (cohort 5 mice) or the retrograde tracing virus rAAV2/Retro-hSyn-mCherry-WPRE-hGH pA (PT-0100, 5.30 × 10^12^ vg/mL, BrainVTA) into mPFC (cohort 5 mice) of WT mice. Trans-synaptic anterograde virus rAAV2/1-hSyn-CRE-WPRE-hGH pA (PT-0136, 1.09 × 10^13^ vg/mL, BrainVTA) was unilaterally injected into CL combined with Cre-dependent virus rAAV2/9-hSyn-DIO-EGFP-WPRE-hGH pA (PT-1103, 5.04 × 10^12^ vg/mL, BrainVTA) in mPFC (cohort 5 mice) to determine the types of downstream receiving neurons in mPFC. After 1 week (CTB-555) or 3 weeks (tracing virus) of transfection, mice were perfused with 0.9% saline, followed by 4% paraformaldehyde (PFA), and images of the CTB-555 or virus signals were analyzed to assess the virus-injected positions. Immunofluorescence images were analyzed and processed using Leica LAS X software (version 3.5.6.21481) to obtain the fluorescence intensity.

For the VTA–CL–mPFC circuit, rAAV2/1-hSyn-CRE-WPRE-hGH pA (1.09 × 10^13^ vg/mL) was unilaterally injected into VTA combined with Cre-dependent virus rAAV-hSyn-DIO-Synaptophysin-EGFP-WPRE-hGH pA (PT-4595, 6 × 10^12^ vg/mL, BrainVTA) in CL (cohort 7 mice). To label ^VTA–^CL^–mPFC^ neurons (cohort 7 mice), rAAV2/1-hSyn-CRE-WPRE-hGH pA (1.09 × 10^13^ vg/mL) was unilaterally injected into VTA, rAAV2/Retro-Ef1α-DIO-FLP-WPRE-hGH pA (PT-0075, 5.45 × 10^12^ vg/mL, BrainVTA) was unilaterally injected into mPFC, and rAAV2/9-hSyn-fDIO-EGFP-WPRE-hGH pA (BC-0477, 5.97 × 10^12^ vg/mL, BrainCase) was unilaterally injected into CL.

### fMOST.

For fMOST imaging, Ai14 mice (cohort 1 mice) were unilaterally injected with rAAV2/Retro-mTH-NLS-Cre-WPRE-SV40 pA (5 × 10^12^ vg/mL) into CL to detect the TH^+^ upstream regions of CL. Furthermore, rAAV2/9-CaMKII-mCherry-WPRE-hGH pA (PT-0108, 5.29 × 10^12^ vg/mL, BrainVTA) was unilaterally injected into CL to detect the CL^Glu^–mPFC pathway (cohort 5 mice). 3 weeks later, male mice were perfused with PBS followed by 4% PFA, and brains were dissected and postfixed in 4% PFA for 24 hours at 4°C. After fixation, the brains were rinsed overnight at 4°C in 0.01 M PBS and subsequently dehydrated in a graded ethanol series (25%, 50%, 70%, and 100%, 2 hours at each concentration) at 4°C. After dehydration, the brains were placed in a brown bottle and infiltrated with 100% LR White mixed Sudan black B resin (BE-L6-05, OEBio) for 9 hours. Finally, the BioMolding LR White Embedding Kit (BE-L6-05, OEBio) was used for polymerization, and the light-proof samples were placed in a constant temperature drying chamber at 37.5°C for 24 hours. The embedded brains were imaged under an fMOST microscope (BioMapping 5000N, OEBio) at a voxel resolution of 0.3 × 0.3 × 2 μm. The collected images were processed using Imaris Viewer software (version 9.0, Oxford Instruments).

### CPP.

The CPP was conducted using the TopScan3D CPP apparatus (CleverSys), which consists of 2 distinct chambers (15 × 15 × 23 cm each) separated by a removable guillotine door. One chamber had walls with vertical stripes and a plaid floor. The other chamber had walls with horizontal stripes and a striped floor. The CPP procedure consisted of 3 phases: the preconditioning test (baseline/pretest, day 0), conditioning (CPP-Training, days 1–8), and postconditioning test (CPP-Test, day 9). The mice were habituated 2 days prior to pretest for 45 minutes per day. Baseline preference (preconditioning test) was determined by allowing mice to freely explore both chambers of the CPP apparatus for 15 minutes. Based on initial chamber preference, the nonpreferred chamber was designated the drug-paired chamber, and the preferred chamber was designated the non–drug-paired chamber. CPP-Training (conditioning) was confined to a non–drug-paired chamber paired with saline (0.2 mL, i.p.) on even days and to a drug-paired chamber paired with METH (1 mg/kg, i.p., China Institute for Food and Drug Control) injection on odd days. After each injection, the mice were confined to the corresponding chamber (non–drug-paired chamber or drug-paired chamber) for 45 minutes. During the CPP-Test (postconditioning test), mice were again placed in the CPP apparatus and allowed to explore the 2 chambers freely without any drug treatment for 15 minutes. The CPP score was the time spent in the drug-paired chamber minus that in the non-drug-paired chamber, and the ΔCPP score was calculated as the CPP-Test CPP score minus the pretest CPP score. The cohort 8′ mice were euthanized before the METH CPP-Test, and the cohort 8 and 9 mice were euthanized after the METH CPP-Test on day 9.

### Immunofluorescence.

Mice were deeply anesthetized with isoflurane and sequentially perfused with 0.9% saline and 4% PFA. The brains were removed, postfixed in 4% PFA at 4°C overnight, and transferred to 30% (w/v) sucrose. The coronal brain sections (30 μm) were continuously sectioned by a cryostat freezing microtome (Leica) and used for immunofluorescence. The sections were washed in PBS 3 times (10 minutes each time), incubated in 0.3% (v/v) Triton X-100 for 30 minutes, blocked with 5% donkey serum for 90 minutes at room temperature, and incubated overnight at 4°C with the following primary antibodies: rabbit anti–c-Fos (1:2,000, RRID: AB_2247211, 2250, Cell Signaling Technology), mouse anti-NeuN (1:800, RRID: AB_2298772, MAB377, Millipore), rabbit anti-CaMKIIα (1:500, RRID: AB_305050, ab52476, Abcam), guinea pig anti–c-Fos (1:3,000, RRID: AB_2905595, 226308, Synaptic Systems), rabbit anti-D1R (1:500, RRID: AB_ 2815007, ET1703-45, HUABio), mouse anti-TH (1:500, RRID: AB_3082539, MA1100, Boster Biological Technology), and mouse anti-GAD67 (1:500, RRID: AB_2938602, MAB5406, Millipore), followed by the corresponding fluorophore-conjugated secondary antibodies for 90 minutes at room temperature. The following secondary antibodies were used: Alexa Fluor 555–labeled donkey anti-rabbit secondary antibody (1:500, RRID: AB_162543, A-31572, Invitrogen), Alexa Fluor 488–labeled donkey anti-rabbit (1:500, RRID: AB_2762833, A32790, Invitrogen), Alexa Fluor 555–labeled donkey anti-mouse secondary antibody (1:500, RRID: AB_2762848, A32773, Invitrogen), Alexa Fluor 488–labeled donkey anti-mouse secondary antibody (1:500, RRID: AB_141607, A-21202, Invitrogen), Alexa Fluor 680–labeled donkey anti-rabbit secondary antibody (1:500, RRID: AB_2762836, A32802, Invitrogen), Alexa Fluor 680–labeled donkey anti-mouse secondary antibody (1:500, RRID: AB_2762831, A32788, Invitrogen), Alexa Fluor 488–labeled donkey anti-guinea pig (1:500, RRID: AB_2736871, ab150185, Abcam), and Alexa Fluor 555–labeled donkey anti-guinea pig (1:500, RRID: AB_3099754, ab150186, Abcam). Fluorescence signals were visualized using either a Leica DMi8 THUNDER Imager 3D tissue microscope or a Leica TCS SP8 confocal laser scanning microscope. The immunofluorescent signal values of D1R receptors on CaMKII^+^ neurons in the CL region of each slice (total value) was calculated by LAS X software (version 3.5.6.21481, Leica). The mean gray value of D1R on each CaMKII^+^ neuron was calculated by dividing the total D1R value by the number of CaMKII^+^ neurons in 1 slice. At least 3 slices from each mouse and 6 mice/group were analyzed.

### Western blot.

Male mice (cohorts 8 and 19) brains were rapidly removed following the CPP-Test and placed in OCT compound (Sakura). The samples were quickly frozen in liquid nitrogen and fixed on a cold sample holder. Brain tissue was coronally cut by a Leica CM1950 cryostat. Bregma +1.54 mm level was determined when the hook-like corpus callosum appeared on both sides of the sample section, followed by a continued cut of 1 mm. A handled biopsy punch (Miltex Instruments) was vertically inserted into the outside of the end of the ventrolateral corpus callosum, and CL tissue was collected and frozen on dry ice. Total protein was extracted from CL in mice using RIPA lysis buffer (Beijing ComWin Biotech Co.) according to the manufacturer’s instructions. Protein samples (15 μg) were separated by 10% and 12% SDS-PAGE and electrophoretically transferred onto PVDF membranes. The transferred membranes were blocked with 5% nonfat dry milk or 5% BCA and 0.1% Tween 20 in 10 mM Tris-HCl (TBST buffer) for 1.5 hours at room temperature and subsequently incubated with the following primary antibodies: D1R (1:1,000, rabbit, RRID: AB_2815007, ET1703-45, HUABio), D2R (1:1,000, rabbit, HA723162, HUABio). The next day, the membranes were washed 3 times in Tris-buffered saline with Tween 20 and incubated with HRP-conjugated secondary antibody goat anti-rabbit (1:5,000, CW0103S, Beijing ComWin Biotech Co.) at room temperature for 1 hour. The blots were visualized by the ECL kit (Beyotime), and the signal was visualized using the Tanon 5200 imaging system. The blots were washed with stripping buffer (Beyotime) to reprobe with other antibodies. GAPDH was used as the loading control. Values for target protein levels were calculated using Image J software (NIH). The relative level of expression of each protein (blot volume) was normalized to GAPDH.

### Fiber photometry.

To observe and record the in vivo activity of CaMKII^+^ neurons (cohort 9 mice) or the release of DA (cohort 3 and 16 mice) in CL, the calcium or DA signaling was recorded during behavioral experiments. Mice were anesthetized with 2% isoflurane in oxygen and fixed in a stereotaxic frame (RWD). A heating pad was used to maintain the core body temperature of the animals at 36°C. 50 nL of the rAAV2/9-CaMKII-GCaMp6m (PT-0111, 5.46 × 10^12^ vg/mL, BrainVTA) or rAAV2/9-CaMKII-DA3h (PT-9098, 5.25 × 10^12^ vg/mL, BrainVTA) virus was injected into CL at a rate of 10 nL/min, and an optical fiber (200 μm outer diameter, 0.37 numerical aperture [NA], AOGUAN Biotech) was placed 100 μm above the viral injection site. The calcium-dependent fluorescence signals were obtained by stimulating cells that transfected with the GCaMp6m virus or DA3h sensor using a 470 nm LED (35–40 μW at the fiber tip), while calcium-independent signals were obtained by stimulating these cells with a 405 nm LED (15–20 μW at the fiber tip). The 470 and 405 nm LEDs were alternated at 40 fps (AOGUAN Biotech), and light emission was recorded using a CMOS camera containing the entire fiber bundle (2 m in length, NA = 0.37, 200 μm core, Thinkertech). The fluorescent analog voltage signals were filtered at 30 Hz and digitized at 100 Hz. The GCaMp6m or DA3h signals (GPCR activation-based sensor for dopamine [3rd generation high sensitivity], BrainVTA) were recorded and analyzed by Thinkertech TrippleColor MultiChannel fiber photometry acquisition software and analysis package (Thinkertech). The raw heatmap data from 1 mouse was merged as a statistical point and normalized using AUC normalization. The AUC represents the integral under the recording duration relative to the corresponding baseline at each trial. Animals with missed injections and fiber implantations were excluded from the study.

During the CPP-Test (cohort 9 and 16 mice), baseline fluorescence (F_0_) was defined as the average signal recorded during the 1 second before the animal’s body fully entered the drug-paired/non–drug-paired chamber from the other side chamber in each recording episode. The real-time fluorescence signal (F) was then recorded for 4 seconds during the CPP-Test period. For each session, ΔF/F was calculated as ΔF/F (%) = (F − F_0_)/F_0_ × 100%.

To record DA signaling in CL combined with chemogenetic activation of the VTA^TH^–CL pathway (cohort 3 mice), WT mice were injected with rAAV2/9-CaMKII-DA3h (5.25 × 10^12^ vg/mL) and rAAV2/Retro-mTH-NLS-Cre-WPRE-SV40 pA (5 × 10^12^ vg/mL) virus into the CL, and rAAV2/9-EF1α-DIO-hM3D(Gq)-mCherry (PT-0042, 5 × 10^12^ vg/mL, BrainVTA) virus was injected into the VTA. The baseline fluorescence signal was recorded for 5 minutes with 1-minute recording and 4-minute intervals (1 session) prior to CNO (2 mg/kg, i.p., Selleck) treatment. The real-time fluorescence signal was recorded for 35 minutes with a 1-minute recording and 4-minute intervals. Animals with missed injections were excluded from the study.

### In vivo chemogenetic manipulation.

For chemogenetic regulation of CL^Glu^ (cohort 10 mice), rAAV2/9-CaMKII-hM4D(Gi)-mCherry-WPRE-hGH pA (PT-0017, 5.32 × 10^12^ vg/mL, BrainVTA) was injected into CL of WT mice.

For chemogenetic regulation of the upstream somas on the VTA^TH^–CL pathway (cohort 13 mice), rAAV2/9-EF1α-DIO-hM4D(Gi)-EGFP-WPREs-hGH pA (PT-0987, 5 × 10^12^ vg/mL, BrainVTA) was injected into VTA, and rAAV2/Retro-mTH-NLS-CRE-WPRE-SV40 pA (5 × 10^12^ vg/mL) and rAAV2/9-CaMKII-mCherry-WPREs-hGH pA (5.29 × 10^12^ vg/mL) were injected into CL.

For chemogenetic regulation of the axon terminals on the VTA^TH^–CL pathway (cohort 14 mice), rAAV2/9-EF1α-DIO-hM4D(Gi)-EGFP-WPREs-hGH pA (5 × 10^12^ vg/mL) and rAAV2/9-mTH-NLS-CRE-WPRE-SV40 pA (5 × 10^12^ vg/mL) were injected into the VTA, and the cannulas (O.D. 0.20 mm, C.C. 3.5 mm, catalog 62104, RWD) were implanted into the CL. 50 nL of saline (vehicle [Veh]) or CNO (10 μM) was injected into CL at a rate of 10 nL/min at 5 minutes prior to the CPP-Test.

For chemogenetic inhibition combined with CNO and pharmacological activation of the VTA^TH^–CL pathway (cohort 17 and 18 mice), rAAV2/9-EF1α-DIO-hM4D(Gi)-EGFP-WPREs-hGH pA (5 × 10^12^ vg/mL) was injected into VTA; cannulas (as described above) were implanted into CL in mice brain and injected with rAAV2/Retro-mTH-NLS-CRE-WPRE-SV40 pA (5 × 10^12^ vg/mL) at a rate of 10 nL/min. After surgery, mice were maintained in home cages for about 3 weeks. The DA hydrochloride (ASL279, MedChemExpress) or D1R agonist (±)-SKF-38393 hydrochloride (SKF-38393, MedChemExpress) was diluted to 10 mM in 0.9% saline by shaking. The onset of this effect required at least 5 minutes, and it persisted for at least 60 minutes in in vivo experiments (51), after which the mixture was aliquoted as a storage solution and stored at –80°C. 50 nL of saline (Veh), DA hydrochloride (1 mg/mL), or SKF (1 mg/mL) mixed with CNO (10 μM) was injected into CL at a rate of 10 nL/min at 5 minutes prior to the CPP-Test.

For chemogenetic regulation of the CL^Glu^–mPFC pathway (cohort 15 mice), rAAV2/9-EF1α-DIO-hM4D(Gi)-WPREs-hGH pA (5 × 10^12^ vg/mL) was injected into CL, and rAAV2/Retro-hSyn-Cre-EGFP-WPRE-hGH pA (PT-1186, 5 × 10^12^ vg/mL, BrainVTA) was injected into mPFC.

For chemogenetic regulation of ^VTA–^CL^–mPFC^ neurons (cohort 12 mice), rAAV2/1-hSyn-CRE-WPRE-hGH pA (1.09 × 10^13^ vg/mL) was injected into VTA, rAAV2/Retro-Ef1α-DIO-FLP-WPRE-hGH pA (5.45 × 10^12^ vg/mL) was injected into mPFC, and rAAV2/9-hSyn-fDIO-hM4D(Gi)-EGFP-WPRE-hGH pA (PT-9137, 2.88 × 10^12^ vg/mL, BrainVTA) was injected into CL.

CNO (2 mg/kg, i.p., Selleck) was administered 30 minutes before the CPP-Test to inhibit the DREADD-expressed neurons by the virus, and saline (0.2 mL) was used as the control vehicle. Animals with missed injections were excluded from the study.

### Local knockdown of D1R on CL^Glu^.

50 nL of rAAV2/9-CaMKII-RNAi-Drd1-EGFP [CaMKIIap-EGFP-MIR155(shDrd1)-SV40 pA, KD group, titer: 8.89 × 10^13^ vg/mL, GeneChem] or rAAV2/9-CaMKII-EGFP [CaMKIIap-EGFP-MIR155(MCS)-SV40 pA, control group, titer: 1 × 10^13^ vg/mL, GeneChem] was injected into CL at a rate of 10 nL/min (cohort 19 mice). After surgery, mice were housed in their home cages for about 3 weeks. Animals with missed injections were excluded from the study.

### Acute brain slice preparation.

Mice were deeply anesthetized with isoflurane (RWD) and perfused with ice-cold cutting solution (in mM: 92 *N*-methyl-d-glutamine, 2.5 KCl, 1.2 NaH_2_PO_4_, 30 NaHCO_3_, 20 HEPES, 0.5 CaCl_2_, 10 MgSO_4_, 25 glucose, 5 sodium ascorbate, 2 thiourea, and 3 sodium pyruvate, pH adjusted to 7.3 with HCl, osmolarity 305, and saturated with 95% O_2_/5% CO_2_). Slices containing the CL or mPFC were cut to 200 μm thickness using a vibratome in 4°C cutting solution. The slices were transferred to 37°C cutting solution and kept for approximately 9 minutes. They were then transferred to oxygenated holding solution (in mM: 86 NaCl, 2.5 KCl, 1.2 NaH_2_PO_4_, 35 NaHCO_3_, 20 HEPES, 2 CaCl_2_, 1 MgSO_4_, 25 glucose, 5 sodium ascorbate, 2 thiourea, and 33 sodium pyruvate, pH adjusted to 7.3, osmolarity 305, and saturated with 95% O_2_/5% CO_2_) to allow for recovery at room temperature for at least 60 minutes before recordings. During electrophysiological recordings, the brain slice was continuously perfused with oxygenated artificial CSF (ACSF; in mM: 119 NaCl, 2.5 KCl, 1 NaH_2_PO_4_, 1.3 MgCl_2_, 2.5 CaCl_2_, 26.2 NaHCO_3_, and 11 glucose, 290 mOsm, saturated with 95% O_2_/5% CO_2_) maintained at 31°C by a solution heater (TC-324C, Warner Instruments).

### Whole-cell patch clamp.

Microelectrodes (3–5 MΩ) were filled with Cs^+^-based internal solution (in mM: 130 CsMeSO_4_, 10 NaCl, 10 EGTA, 4 Mg-ATP, 0.3 Na-GTP, and 10 HEPES, pH 7.25–7.30, 290 mOsm), and voltage-clamp protocols were run at –70 mV.

To verify the connectivity between VTA and CL (cohort 4 mice) or CL and mPFC (cohort 6 mice), rAAV2/9-EF1α-DIO-hChR2(H134R)-mCherry-WPRE-hGH pA (PT-0002, 5.05 × 10^12^ vg/mL, BrainVTA) and rAAV2/9-mTH-NLS-CRE-WPRE-SV40 pA (5.38 × 10^12^ vg/mL) were injected into VTA; rAAV2/9-CaMKII-hChR2(H134R)-mCherry-WPRE-hGH pA (PT-0297, 5.45 × 10^12^ vg/mL, BrainVTA) was injected into CL. VTA^TH^ or CaMKII^+^ neurons in CL were identified by mCherry, and the APs induced with 300 pA current injection of CL neurons or light-evoked EPSCs of mPFC neurons in voltage clamp were recorded. Optical stimulation was applied in VTA^TH^ terminals in CL (473 nm, 5 mW; 10 Hz) or CL^Glu^ terminals in mPFC (473 nm, 5 mW; 20 Hz) expressing ChR2. SCH-23390 (10 μM) was used in the recording ACSF solution to verify whether D1R is required for the response evoked by the VTA^TH^–CL pathway stimulation. TTX (1 μM), 4-AP (100 μM), NBQX (10 μM), and AP5 (20 μM) were used in the recording ACSF solution to verify whether the CL^Glu^–mPFC pathway has a monosynaptic connection.

To assess the excitability of ^VTA–^CL^–mPFC^ neurons in the METH CPP behavior, recordings were performed under current-clamp mode (cohort 11 mice). Whole-cell patch clamp microelectrodes (3–5 MΩ) were filled with potassium internal solution. For measurements of neuronal excitability, the AP was determined following current injections at different intensities (0–200 pA with a 10 pA increment) at –70 mV.

To confirm the functionality of expressed hM3Dq and hM4Di, the hM3Dq- and hM4Di-labeled neurons were recorded in whole-cell patch clamp mode, and the sAPs were determined before and after bath application of CNO (10 μM, 5–10 minutes).

All the light stimulations were applied using a QAXK 473 nm laser (Thinkertech). All signals were filtered at 4 kHz, amplified 5× using a MultiClamp 700B amplifier (Molecular Devices), and digitized at 10 kHz with a Digidata 1550B analog-to-digital converter (Molecular Devices). All data were analyzed with Clampfit 10.6 software (Molecular Devices).

### Sholl analysis and dendritic spine analysis.

Coronal sections (60 μm thick) were processed for imaging. Confocal *Z*-stacks of EGFP cells were taken from each CL (from 1.10 to 0.38 mm anterior to bregma) section per mouse (cohort 11 mice). The Fiji v2.0.0 stitching plug-in was used to conduct Sholl analysis, and the density of dendritic spines was determined. The center of the somas was defined as the center of all concentric circles.

### Statistics.

Statistical analysis was carried out using GraphPad Prism 8.0.2 software. All data are presented as the mean ± SEM. The data were analyzed by unpaired 2-tailed Student’s *t* test, paired 2-tailed Student’s *t* test, and 2-way ANOVA with Šidák’s multiple-comparison test where appropriate. All statistical significance was set as *P* < 0.05.

### Study approval.

All procedures were carried out in accordance with the NIH Guide for the Care and Use of Laboratory Animals and approved by the IACUC at Nanjing University of Chinese Medicine.

### Data availability.

The data that support the findings of this study are available from the corresponding author upon reasonable request. Raw data are available in the Supporting Data Values file.

## Author contributions

ZZ and YH performed the experiments and collected the data. ZZ and YF performed the data curation and analysis. YL and QF conducted data analysis and behavioral experiments. ZZ, YF, and XG prepared figures and wrote the first draft. HYK edited the manuscript. XG conceptualized the overall concept and supervised the project. The order of the co–first authors was assigned based on their specific contributions and efforts to the study.

## Funding support

- National Natural Science Foundation of China (82271531 to XG and 82405570 to YF).
- Natural Science Foundation of Jiangsu Province of China (BK20240726 to YF).
- Traditional Chinese Medicine Technology Development Project of Jiangsu Province (ZD202302 to XG and MS2022003 to YF).

## Supplementary Material

Supplemental data

Unedited blot and gel images

Supporting data values

## Version 1 01/15/2026

In-Press Preview

## Version 2 03/02/2026

Electronic publication
