# Corrigendum to A midbrain–cortical circuit mediated by a claustrum neuronal ensemble orchestrates drug-paired context memory processing

**DOI:** 10.1172/JCI211416

**Published:** 2026-09-15

**Authors:** Ziheng Zhao, Yuhong He, Yang Liu, Quying Feng, Hee Young Kim, Yu Fan, Xiaowei Guan

Original citation: *J Clin Invest*. 2026;136(5):e196944. https://doi.org/10.1172/JCI196944

Citation for this corrigendum: *J Clin Invest*. 2026;136(18):e211416. https://doi.org/10.1172/JCI211416

After publication of this article, the authors became aware of data entry errors for Supplemental Figure 2H. Specifically, five of the raw values for the Saline‑CNO (*n* = 6) group were incorrect and were identical to raw values for the METH‑CNO (*n* = 6) group. The supplemental material and Supporting Data Values file have been updated online with the correct values.

The authors regret the error.

## Version 1 09/15/2026

Electronic publication

