# Supplementary material for: A midbrain–cortical circuit mediated by a claustrum neuronal ensemble orchestrates drug-paired context memory processing
Source: J Clin Invest. 2026 Jan 15;136(5):e196944. doi: 10.1172/JCI196944 (PMC12948420; doi:10.1172/JCI196944)
Supplement: Supplemental data [file jci-136-196944-s128.pdf]

SUPPLEMENTARY INFORMATION

A midbrain–cortical circuit mediated by claustrum neuronal ensemble orchestrates drug-paired context memory processing

Ziheng Zhao [1,#], Yuhong He [1,#], Yang Liu [1], Quying Feng [1], Hee Young Kim [2], Yu Fan [1,✉], Xiaowei Guan [1,✉]

[1] Department of Human Anatomy and Histoembryology, Nanjing University of Chinese Medicine, Nanjing 210023, China
[2] Department of Physiology, Yonsei University College of Medicine, Seoul 03722, South Korea

Address correspondence to: Xiaowei Guan, Department of Human Anatomy and Histoembryology, Nanjing University of Chinese Medicine, 138 Xianlin Rd, Nanjing 210023, China. Phone number: +86-25-85811969. Email: guanxw918@njucm.edu.cn. Or to: Yu Fan, Department of Human Anatomy and Histoembryology, Nanjing University of Chinese Medicine, 138 Xianlin Rd, Nanjing 210023, China. Phone number: +86-25-85811920. Email: yfan@njucm.edu.cn.

[#]Authorship note: Ziheng Zhao and Yuhong He contributed equally to this work.

[✉]Corresponding author.

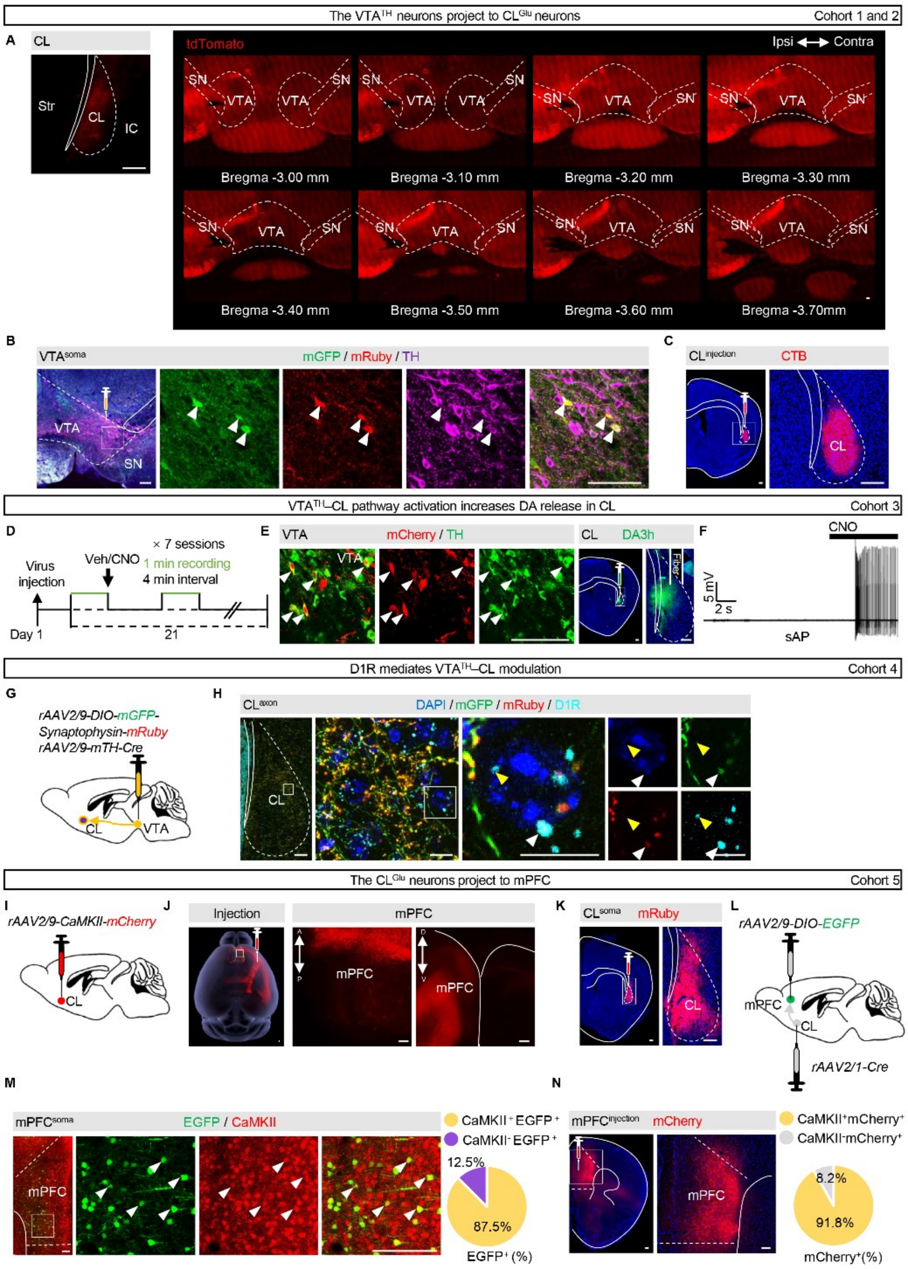

Supplemental Figure 1. There exist a VTATH–CL^{Glu}–mPFC circuit and the $^{VTA-}$CL^{-mPFC} neuronal ensemble. (**A**) The representative images of virus injection and tdTomato^{+} neurons in VTA. Str, striatum; IC, insular cortex. (**B**) Representative images injection and mGFP^{+}mRuby^{+}TH^{+} neurons in VTA. (**C**) Representative images of injection in CL. (**D**) Experimental design and timeline. (**E**) Representative images injection and fiber implantation in CL and mCherry^{+}TH^{+} neurons in VTA. (**F**) Representative voltage traces recorded from hM3Dq-expressing neuron during application of CNO. (**G**) Schematic diagram of anterograde viral transfection (same with Figure 1C). (**H**) Representative images of D1R on presynaptic membrane (the white arrow) and postsynaptic membrane (the yellow arrow). (**I**) fMOST schematic diagram of viral transfection. (**J**) Representative images of axon terminals. (**K**) Representative images of virus injection in CL. (**L**) Schematic diagram of anterograde viral transfection. (**M**) Representative images of trans-synaptic virus injection and the proportion of EGFP^{+}CaMKII^{+} neurons in EGFP^{+} neurons of mPFC. (**N**) Representative images of retrograde virus injection in mPFC and the proportion of CaMKII^{+}mCherry^{+} or CaMKII^{-}mCherry^{+} neurons in mCherry^{+} neurons of CL. Scale bar, 100 μm (**A**, **B**, **C**, **E**, **H**, **J**, **K, M** and **N**) or 10 μm (**H**),

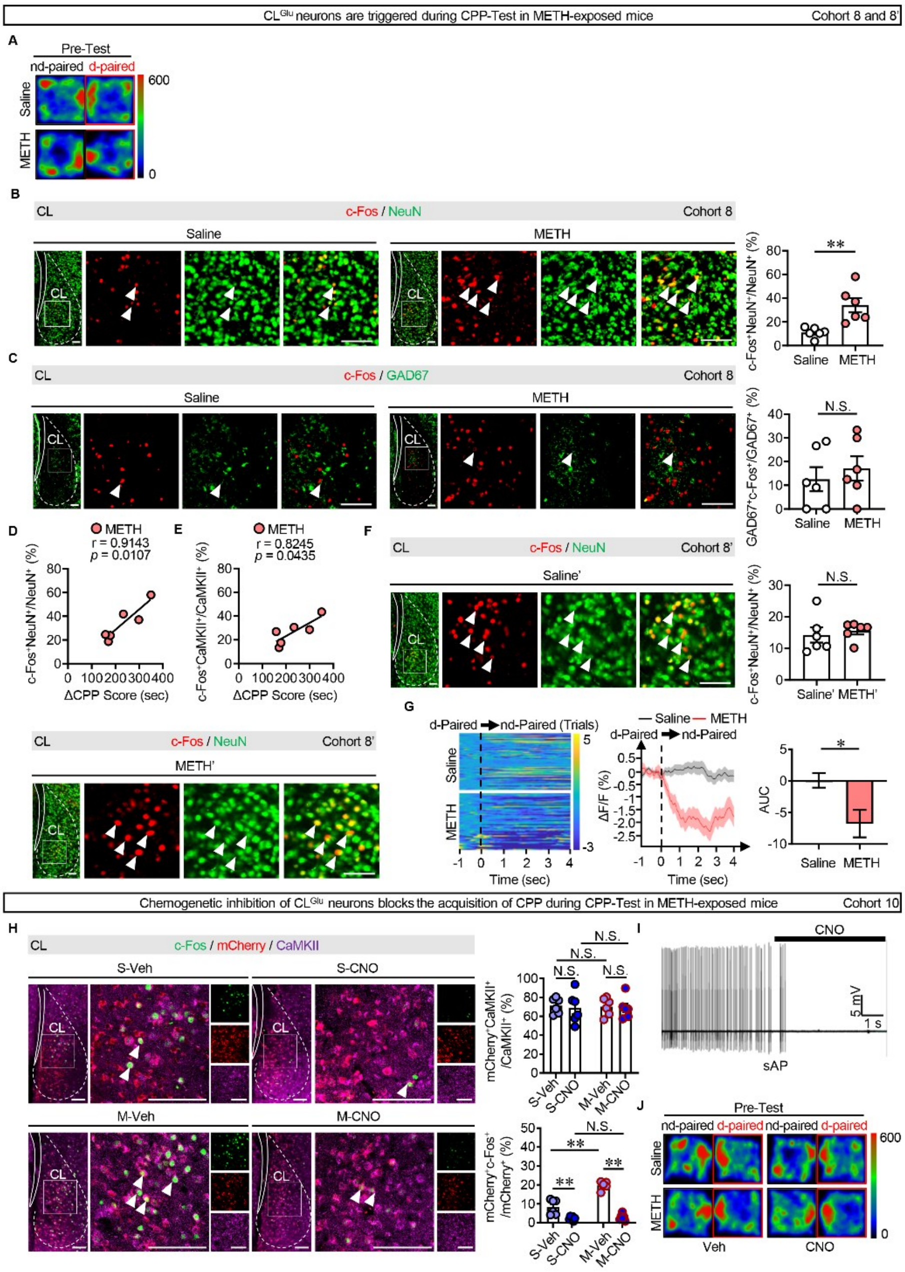

Supplemental Figure 2. The CL^{Glu} neurons are involved in the retrieval of METH-induced reward memory. (**A**) Representative heatmap of time spent in CPP apparatus during CPP Pre-Test. (**B**) Immunofluorescence for c-Fos^{+}NeuN^{+} neurons in CL following METH CPP-Test. The percentage of c-Fos^{+}NeuN^{+} neurons in CL. n = 6 mice/group. (**C**) Immunofluorescence for c-Fos^{+}GAD67^{+} neurons in CL following METH CPP-Test. The percentage of c-Fos^{+}GAD67^{+} neurons in CL^{GABA}. n = 6 mice/group. (**D**) The correlation analysis of CL neurons activity with ΔCPP Score of METH-exposed mice. n = 6 mice. (**E**) The correlation analysis of CL CaMKII^{+} neurons activity with ΔCPP Score of METH-exposed mice. n = 6 mice. (**F**) Immunofluorescence for c-Fos^{+}NeuN^{+} neurons in CL before METH CPP-Test. The percentage of c-Fos^{+}NeuN^{+} neurons in CL. n = 6 mice/group. (**G**) Heatmap of GCaMp6m fluorescence (left), quantification (middle) and AUC (right) of ΔF/F in CL. The AUC of ΔF/F. n = 6 mice/group. (**H**) Immunofluorescence for c-Fos^{+}mCherry^{+}CaMKII^{+} neurons in CL following METH CPP-Test. The percentage of mCherry^{+}CaMKII^{+} neurons in CL^{Glu} and c-Fos^{+}mCherry^{+} neurons in mCherry^{+} neurons of CL. n = 6 mice/group. (**I**) Representative voltage traces recorded from hM4Di-expressing neuron during application of CNO. (**J**) Representative heatmap of time spent in CPP apparatus during CPP Pre-Test. N.S., $p > 0.05$, $*p < 0.05$, $**p < 0.01$. Two-way ANOVA with Sidak's multiple-comparisons test (**H**), two-tailed unpaired t test (**B**, **C**, **F** and **G**). Scale bar, 100 μm (**B**, **C**, **F** and **H**).

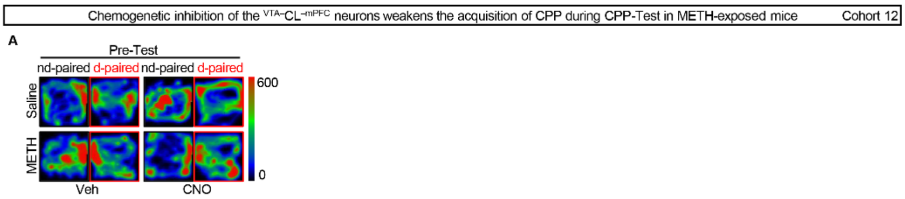

Supplemental Figure 3. The $^{VTA-}CL^{-mPFC}$ neuronal ensemble mediates the retrieval of METH-induced reward memory. (**A**) Representative heatmap of time spent in CPP apparatus during CPP Pre-Test.

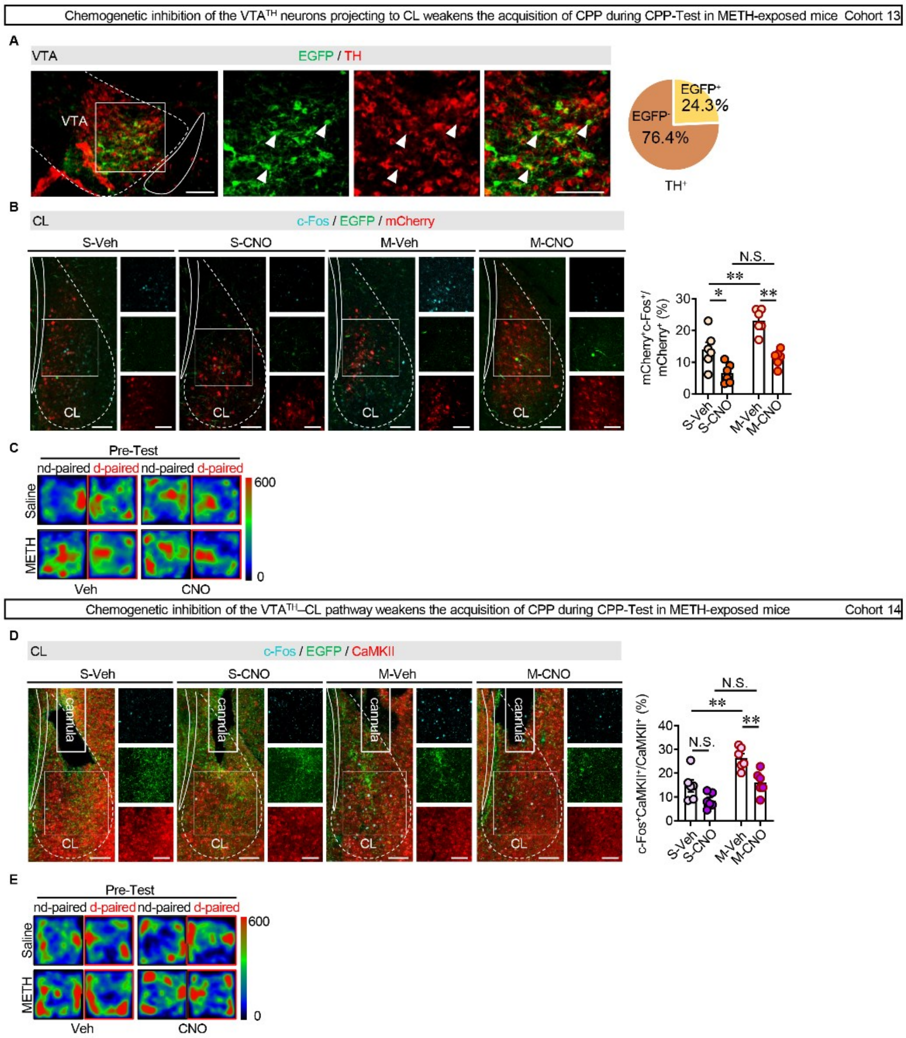

Supplemental Figure 4. VTATH–CL pathway contributes to the retrieval of METH-induced reward memory. (**A**) Representative images of EFGP$^+$TH$^+$ neurons in VTA (left panel) and co-localization percentage of EFGP$^+$TH$^+$ neurons in TH$^+$ neurons (right panel). (**B**) Immunofluorescence for c-Fos$^+$mCherry$^+$ neurons in CL following METH CPP-Test. The percentage of c-Fos$^+$mCherry$^+$ neurons in mCherry$^+$ neurons of CL. n = 6 mice/group. (**C**) Representative heatmap of time spent in CPP apparatus during CPP Pre-Test. (**D**) Immunofluorescence for c-Fos$^+$CaMKII$^+$ neurons in CL following METH CPP-Test. The percentage of c-Fos$^+$CaMKII$^+$ neurons in CL^{Glu}. n = 6 mice/group. (**E**) Representative heatmap of time spent in CPP apparatus during CPP Pre-Test. N.S., $p > 0.05$, *$p < 0.05$, **$p < 0.01$. Two-way ANOVA with Sidak's multiple-comparisons test (**B** and **D**). Scale bar, 100 μm (**A**, **B**, and **D**).

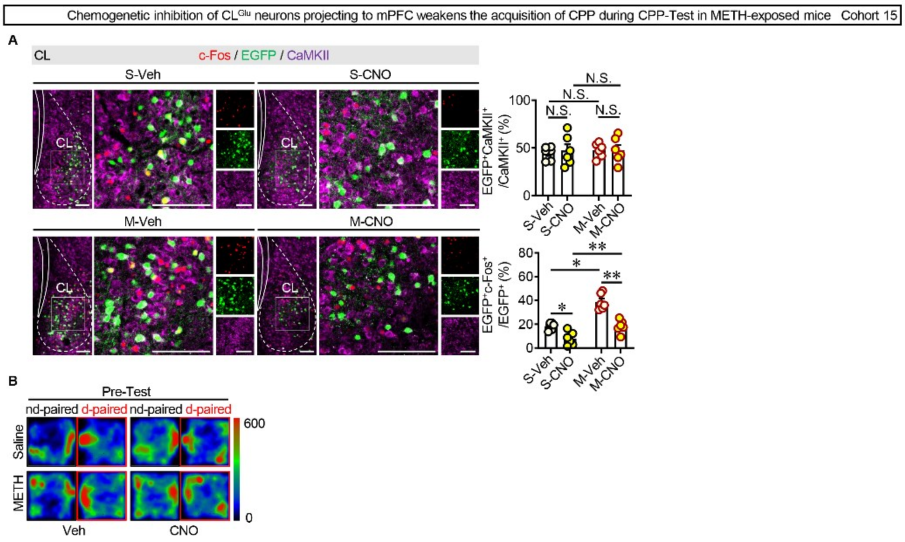

Supplemental Figure 5. CL^{Glu}–mPFC pathway contributes to the retrieval of METH-induced reward memory. (**A**) Immunofluorescence for c-Fos$^+$EGFP$^+$CaMKII$^+$ neurons in CL following METH CPP-Test. Upper panel, the percentage of virus transfected efficiency in CaMKII$^+$ neurons. Lower panel, the percentage of c-Fos$^+$EGFP$^+$ neurons in EGFP$^+$ neurons of CL. n = 6 mice/group. (**B**) Representative heatmap of time spent in CPP apparatus during CPP Pre-Test. N.S., $p > 0.05$, *$p < 0.05$, **$p < 0.01$. Two-way ANOVA with Sidak's multiple-comparisons test (**A**). Scale bar, 100 μm (**A**).

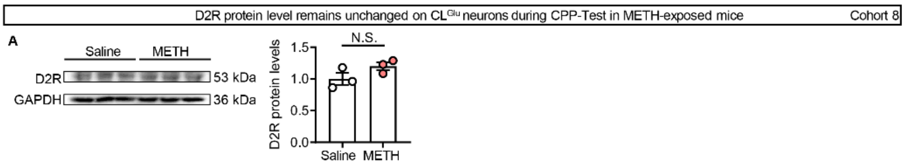

Supplemental Figure 6. The DA release and D1R levels on CL^{Glu} neurons increase during the retrieval of METH-induced reward memory. (**A**) The protein levels of D2R following METH CPP-Test. n = 3 mice/group. N.S., $p > 0.05$. Two-tailed unpaired t test (**A**).

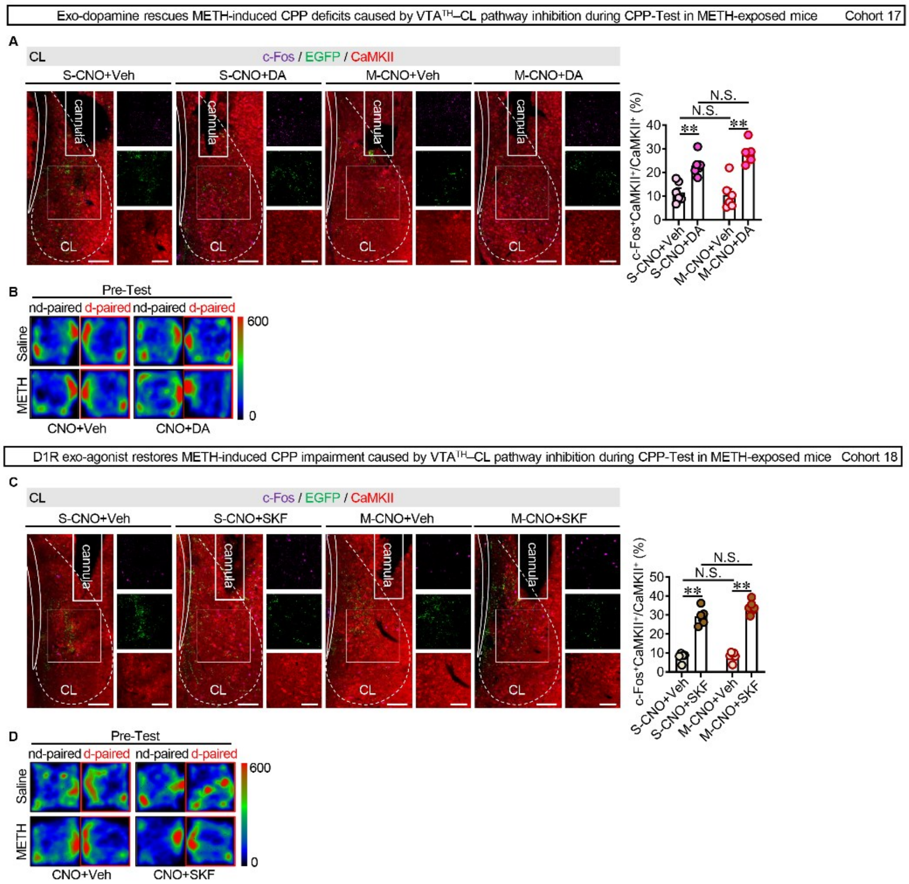

Supplemental Figure 7. DA signaling along the VTATH–CL pathway is crucial for the retrieval of METH-induced reward memory. (**A**) Immunofluorescence for c-Fos$^+$CaMKII$^+$ neurons in CL following METH CPP-Test. The percentage of c-Fos$^+$CaMKII$^+$ neurons in CL^{Glu}. n = 6 mice/group. (**B**) Representative heatmap of time spent in CPP apparatus during CPP Pre-Test. (**C**) Immunofluorescence for c-Fos$^+$CaMKII$^+$ neurons in CL following METH CPP-Test. The percentage of c-Fos$^+$CaMKII$^+$ neurons in CL^{Glu}. S-CNO+Veh/S-CNO+SKF group, n = 5 mice/group; M-CNO+Veh/M-CNO+SKF group, n = 6 mice/group. (**D**) Representative heatmap of time spent in CPP apparatus during CPP Pre-Test. N.S., $p > 0.05$, $^{**}p < 0.01$. Two-way ANOVA with Sidak's multiple-comparisons test (**A** and **C**). Scale bar, 100 μm (**A** and **C**).

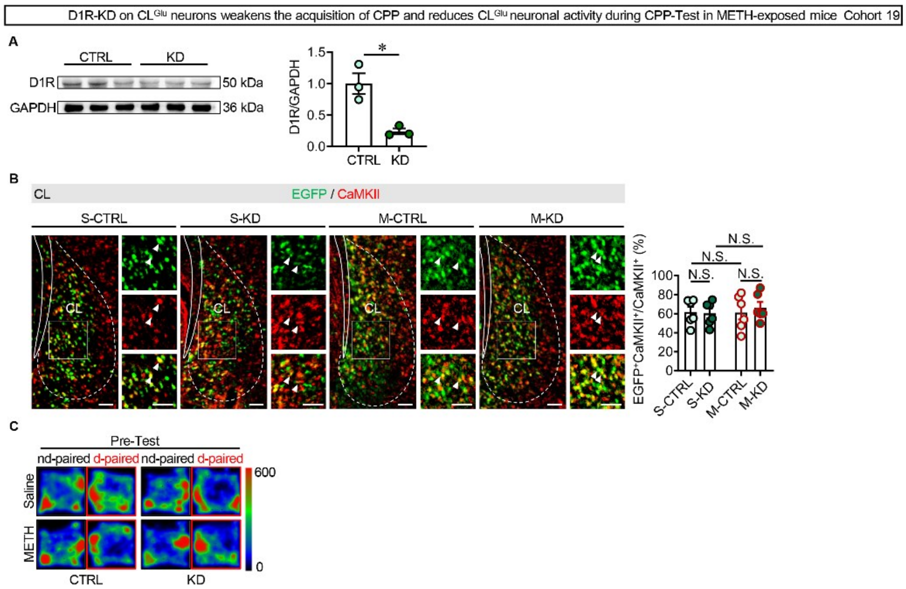

Supplemental Figure 8. D1R on CL^{Glu} neurons modulates the retrieval of METH-induced reward memory. (**A**) The protein levels of D1R. n = 3 mice/group (**B**) Immunofluorescence for EGFP^{+}CaMKII^{+} neurons in CL following METH CPP-Test. The percentage of EGFP^{+}CaMKII^{+} neurons in CL^{Glu} n = 6 mice/group. (**C**) Representative heatmap of time spent in CPP apparatus during CPP Pre-Test. N.S., *p* > 0.05, **p* < 0.05. Two-way ANOVA with Sidak's multiple-comparisons test (**B**), two-tailed unpaired t test (**A**). Scale bar, 100 μm (**B**).
