# Supplementary material for: A midbrain–cortical circuit mediated by a claustrum neuronal ensemble orchestrates drug-paired context memory processing
Source: J Clin Invest. 2026 Jan 15;136(5):e196944. doi: 10.1172/JCI196944 (PMC12948420; doi:10.1172/JCI196944)
Supplement: Unedited blot and gel images [file jci-136-196944-s129.pdf]

Full unedited blot for Figure 6E

Original PVDF	Original ECL blots	Figure
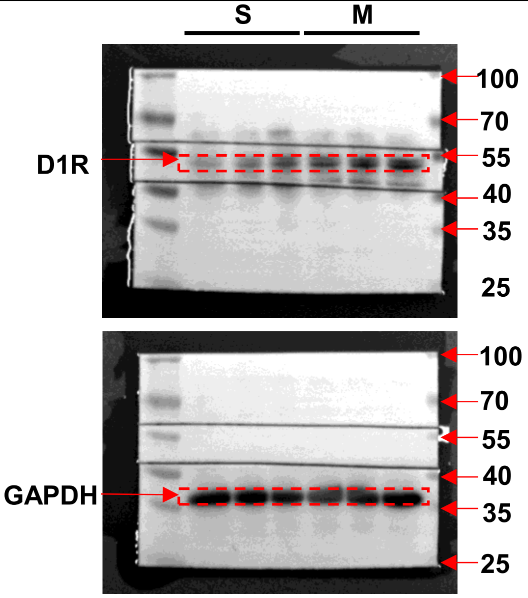	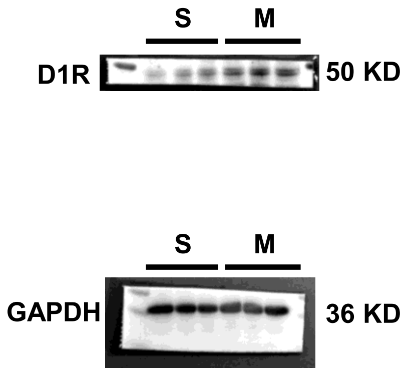	Full unedited blot for Figure 6E

Full unedited blot for Supplemental Figure 6A

Original PVDF	Original ECL blots	Figure
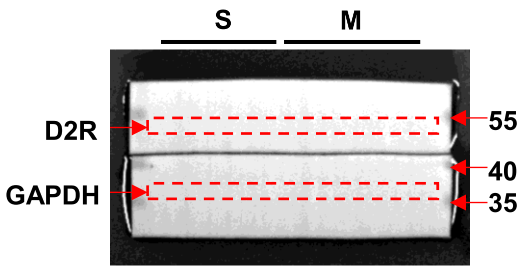	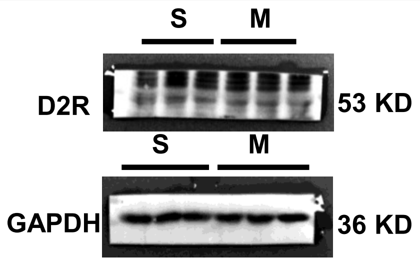	Full unedited blot for Supplemental Figure 6A

Full unedited blot for Supplemental Figure 8A

Original PVDF	Original ECL blots	Figure
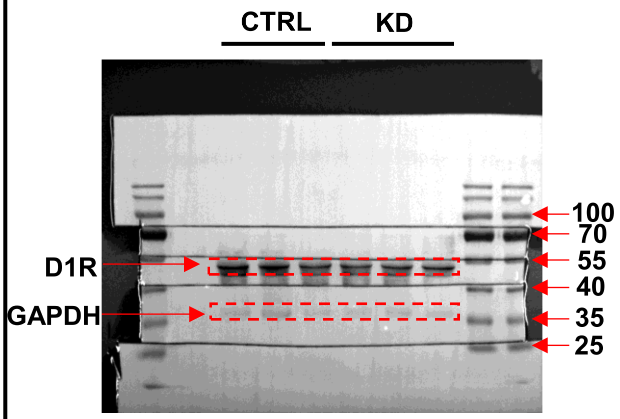	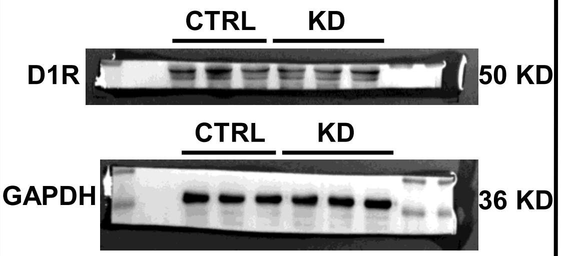	Full unedited blot for Supplemental Figure 8A
